# TRPM8 inhibits substance P release from primary sensory neurons via PKA/GSK-3beta to protect colonic epithelium in colitis

**DOI:** 10.1038/s41419-024-06480-5

**Published:** 2024-01-27

**Authors:** Zehua Zhang, Xiaohan Yan, Le Kang, Zhuyun Leng, Yingjie Ji, Shuangzhu Yang, Xiaojing Du, Kang Fang, Zeyu Wang, Zhaoxing Li, Mingchuang Sun, Ziying Zhao, Anqi Feng, Zhukai Chen, Shihan Zhang, Dong Wan, Tao Chen, Meidong Xu

**Affiliations:** 1 Endoscopy Center, Department of Gastroenterology, Shanghai East Hospital, Tongji University School of Medicine, Shanghai, China; 2 Department of Gastroenterology, Changhai Hospital, Naval Medical University, Shanghai, China

**Keywords:** Somatic system, Ulcerative colitis

## Abstract

Transient receptor potential melastatin 8 (TRPM8) is a cold sensory receptor in primary sensory neurons that regulates various neuronal functions. Substance P (SP) is a pro-inflammatory neuropeptide secreted by the neurons, and it aggravates colitis. However, the regulatory role of TRPM8 in SP release is still unclear. Our study aimed to investigate TRPM8’s role in SP release from primary sensory neurons during colitis and clarify the effect of SP on colonic epithelium. We analyzed inflammatory bowel disease patients’ data from the Gene Expression Omnibus dataset. Dextran sulfate sodium (DSS, 2.5%)-induced colitis in mice, mouse dorsal root ganglion (DRG) neurons, ND7/23 cell line, and mouse or human colonic organoids were used for this experiment. Our study found that TRPM8, TAC1 and WNT3A expression were significantly correlated with the severity of ulcerative colitis in patients and DSS-induced colitis in mice. The TRPM8 agonist (menthol) and the SP receptor antagonist (Aprepitant) can attenuate colitis in mice, but the effects were not additive. Menthol promoted calcium ion influx in mouse DRG neurons and inhibited the combination and phosphorylation of PKAca from the cAMP signaling pathway and GSK-3β from the Wnt/β-catenin signaling pathway, thereby inhibiting the effect of Wnt3a-driven β-catenin on promoting SP release in ND7/23 cells. Long-term stimulation with SP inhibited proliferation and enhanced apoptosis in both mouse and human colonic organoids. Conclusively, TRPM8 inhibits SP release from primary sensory neurons by inhibiting the interaction between PKAca and GSK-3β, thereby inhibiting the role of SP in promoting colonic epithelial apoptosis and relieving colitis.

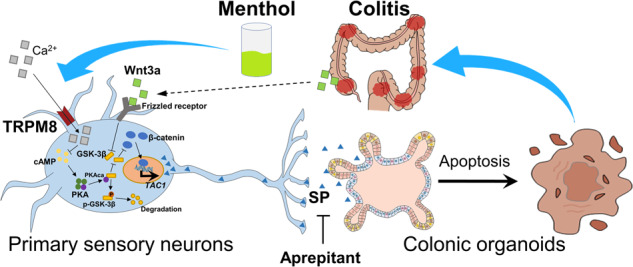

## Introduction

Inflammatory bowel disease (IBD) is a chronic inflammatory disease of the intestine with unknown etiology that includes ulcerative colitis (UC) and Crohn’s disease [1]. Recently, the nervous system has been considered to play an important regulatory role in the pathogenesis of IBD [2]. A previous clinical study showed that rectal sensation of bowel distension is reduced in patients with UC [3]. Moreover, surgical cutting of the colonic sensory nerves led to aggravation of 2,4,6-trinitrobenzene sulfonic acid solution (TNBS)-induced colitis in rats [4]. Transient receptor potential (TRP) channels sense temperature changes and nociceptive stimuli and subsequently induce extracellular calcium ion influx [5, 6]. TRP melastatin 8 (TRPM8) is a non-selective ion channel that senses cold (<28 °C), and can be activated by the compounds that induce cold sensation, such as menthol and icilin [7, 8]. TRPM8 is mainly expressed in neurons, especially in primary sensory neurons that are responsible for sensing temperature changes and nociceptive stimuli [8]. Previous study revealed that TRPM8 attenuates TNBS- or dextran sulfate sodium (DSS)-induced colitis in mice [9]. Another experimental study found that the activation of TRPM8 inhibits lipopolysaccharide (LPS)-induced tumor necrosis factor-alpha (TNF-α) secretion and promotes interleukin-10 (IL-10) secretion by suppressing the mitogen-activated protein kinase (MAPK) pathway in murine macrophages [10]. TRPM8 is highly co-expressed with substance P (SP) in the distal colonic nerve [11]. SP is considered to modulate the immune system and exacerbate intestinal mucosal inflammation during active IBD [12]. Previous studies have demonstrated that the protein level of SP and the expression of its neurokinin-1 receptor (NK-1R) are elevated in the colorectum of IBD patients and are closely related to disease severity [13, 14].

TRPM8 is one of the most important temperature and nociceptive receptors in sensory neurons, and it has the ability to alleviate colitis. However, its effect on colonic primary sensory neurons remains unclear, and may be an important mechanism by which TRPM8 alleviates colitis. Our study elucidates TRPM8 modulatory effects on SP secretion from primary sensory neurons and the effects of SP on colonic epithelial cells, and proposes that menthol and the SP receptor antagonist Aprepitant may serve as therapeutic options for IBD [15].

## Results

### The role of TRPM8 and TAC1 in colitis

**Fig. 1:**
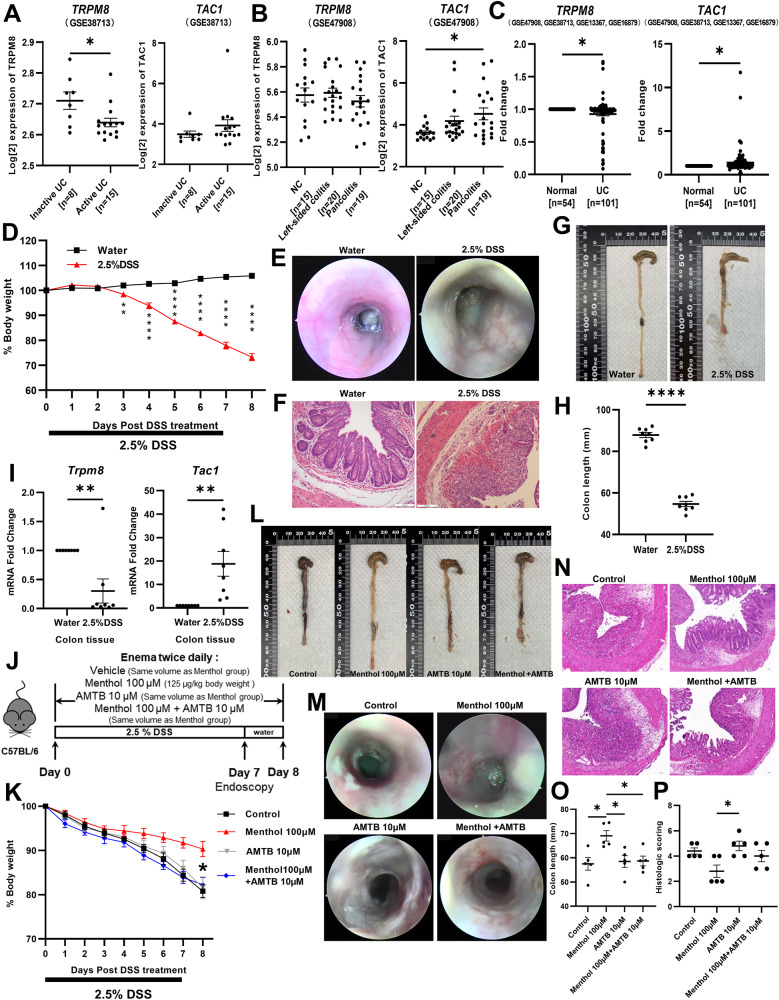
The role of TRPM8 and TAC1 in colitis. A The mRNA expression of *TRPM8* and *TAC1* in inactive UC tissues (n = 8) and active UC tissues (n = 15) indicated by data from GSE38713, and **B** in normal tissues (n = 15), left-sided colitis tissues (*n* = 20) and pancolitis tissues (*n* = 19) indicated by data from GSE47908. **C** The opposite expression of *TRPM8* and *TAC1* in normal tissues (*n* = 54) and UC tissues (n = 101) indicated by data from GSE38713, GSE47908, GSE13367, and GSE16879. **D** The body weight comparisons between the DSS-induced colitis group and the water group (n = 8). **E** The endoscopic appearance and **F** H&E staining pictures of DSS-induced colitis group and the water group. **G**, **H** The comparison of the colon lengths between the two groups of mice on day 8 (n = 8). **I** *Trpm8* and *Tac1* were oppositely expressed in the colon tissues of the DSS-induced colitis group and the water group (n = 8). **J** Grouping method, drug dosages and examination time points of animal experiments. **K** The body weight curves were drawn for four groups of colitis mice (n = 5). **L** The photographs of colons with scale bars in four groups of mice and **O** the summary graph (n = 5). **M** The colons of four groups mice were subjected by endoscopy. **N** Colonic H&E staining pictures of four groups mice and **P** the histological scoring graph (n = 5). *P < 0.05; **P < 0.01; ****P < 0.0001.

To clarify *TRPM8* and *TAC1* (encodes SP) expression in patients with UC, we analyzed data from the Gene Expression Omnibus (GEO) dataset. In GSE38713, *TRPM8* expression was significantly lower in patients with active UC than in those with inactive UC, whereas *TAC1* expression showed the opposite trend (Fig. 1A). Moreover, another study of UC (GSE47908) revealed that *TAC1* expression was significantly higher in patients with pancolitis than in those with left-sided colitis and normal individuals, whereas *TRPM8* showed the opposite trend (Fig. 1B). To eliminate detection differences, we used the expression value of normal individuals as the standard to perform a fold-change analysis of the datasets (GSE38713, GSE47908, GSE13367, and GSE16879). The analysis showed that TRPM8 and TAC1 have opposite expression in UC: *TRPM8* and *TAC1* expression were significantly decreased and increased, respectively, in patients with UC (Fig. 1C), which may be positively related to the severity of UC. The expression of *TRPM8* and *TAC1* in GSE13367 and GSE16879 has been presented in Supplementary Fig. 1. Furthermore, we investigated these trends in experimental colitis models. Compared with control mice, DSS-treated mice had significantly reduced body weight and shortened colon length, and both endoscopic and pathological changes revealed more severe mucosal damage (Fig. 1D–H). Subsequently, we examined gene expression in the two groups. The results showed that *Trpm8* and *Tac1* expression were significantly decreased and increased, respectively in the DSS-induced colitis group (Fig. 1I). The changes in the expression of these two genes were consistent with the data from IBD patients.

To elucidate the TRPM8 role in colitis, we administered TRPM8 agonist (menthol) and/or antagonist (AMTB) via enema to the DSS-treated mice (Fig. 1J). Compared to the other three groups, the menthol-treated group developed only mild signs of colitis. There was significantly less body weight loss (Fig. 1K) and shorter changes in colon length (Fig. 1L, O) than in the other groups. In addition, endoscopic and histopathological analyses of colonic tissues using H&E staining showed that menthol treatment was accompanied by decreased inflammatory cell infiltration and less destruction of the mucosal epithelial layer in response to DSS treatment (Fig. 1M, N, P). The above results showed that the TRPM8 agonist, menthol, significantly attenuated DSS-induced colitis in mice. Moreover, in the menthol-treated group, *Trpm8* expression was significantly increased (Supplementary Fig. 2C), and the expression of inflammatory cytokines such as *Tnf*, *Ifng*, and *Il23a* was significantly decreased (Supplementary Fig. 2A). The serum level of the pro-inflammatory factor, TNF-α, was decreased in the menthol-treated group (Supplementary Fig. 2B). *Tac1* gene expression in the colon and serum SP level were significantly decreased in the menthol-treated group (Supplementary Fig. 2D, E). To verify the effect of TRPM8 on SP release from the isolated whole colon, we performed SP release experiments (Supplementary Fig. 3A) [16]. The results showed that menthol significantly inhibited the release of colonic SP, whereas the application of AMTB had the opposite effect (Supplementary Fig. 3B, C).

### The activation of TRPM8 inhibits SP release from primary sensory neurons

**Fig. 2:**
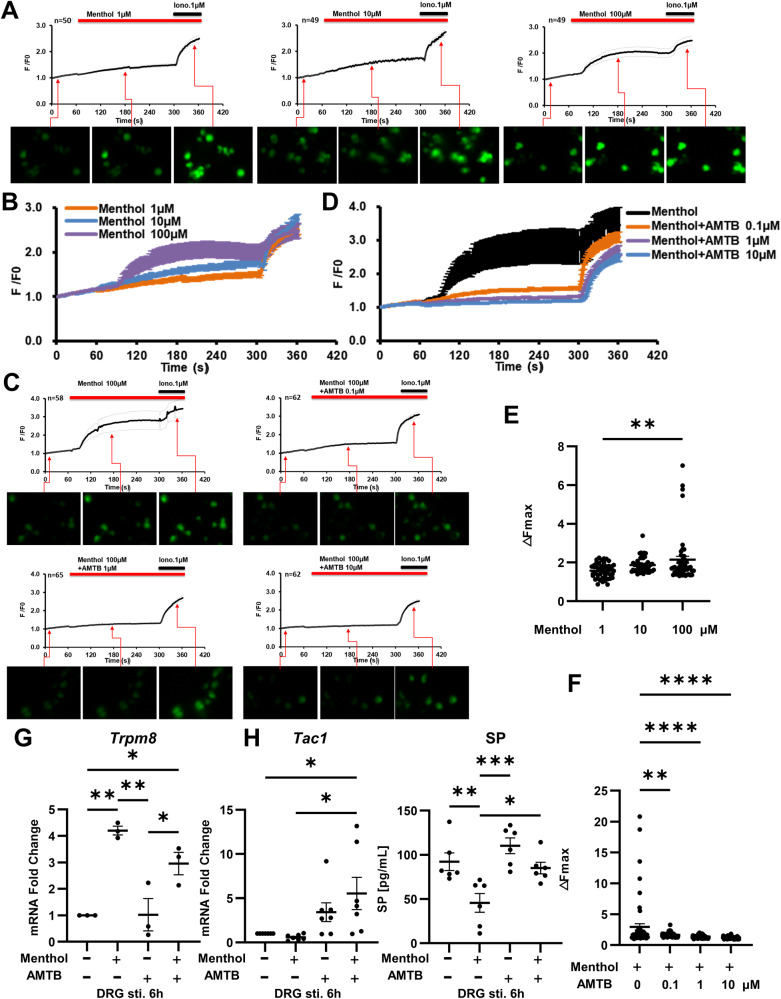
Menthol induces calcium influx and inhibits SP release in DRG neurons. **A** The calcium influx induced by different concentrations of menthol on DRG neurons were plotted as curves. Iono. (ionomycin 1 μM) was used to test the availability of DRG neurons, n indicated the number of neurons. **B** The calcium influx curves induced by different concentrations of menthol on DRG neurons were combined for comparison. **E** The maximum calcium influx intensities in DRG neurons induced by different concentrations of menthol were compared. **C** The calcium influx induced by different concentrations of AMTB and 100 μM menthol on DRG neurons were plotted as curves, **D** the calcium influx curves were combined, and **F** the maximum calcium influx intensity was compared, n indicated the number of neurons. **G** The expression of *Trpm8* in DRG neurons stimulated by menthol and/or AMTB for 6 h (n = 3). **H** The expression of *Tac1* (n = 3) and the protein levels of SP (n = 6) in DRG neurons and supernatants after stimulated by menthol and/or AMTB for 6 h. *P < 0.05; **P < 0.01; ***P < 0.001; ****P < 0.0001.

TRPM8 is highly expressed on primary sensory neurons located in the dorsal root ganglion (DRG) [8]. We examined the calcium ion influx in the DRG neurons induced by the different concentrations of menthol. The results showed that menthol induced calcium ion influx in DRG neurons in a concentration-dependent manner (Fig. 2A, B), with 100 μM menthol having the strongest effect (Fig. 2B, E). Next, we examined the inhibitory effects of different concentrations of AMTB on 100 μM menthol-induced calcium ion influx in DRG neurons. The results showed that the inhibitory effect of AMTB on menthol-induced calcium ion influx was concentration-dependent (Fig. 2C, D), with 10 μM AMTB showing the strongest inhibitory effect (Fig. 2D, F).

Previous single-cell sequencing studies have shown that *Trpm8* and *Tac1* are co-expressed in the same cell population of DRG neurons [17]. Based on the most effective concentrations in the above results, we used menthol and/or AMTB to stimulate DRG neurons for 6 h. The results showed that menthol significantly promoted *Trpm8* expression (Fig. 2G) and inhibited *Tac1* expression in DRG neurons (Fig. 2H). Moreover, menthol stimulation markedly reduced the level of SP in the supernatants (Fig. 2H). Taken together, our results suggested that TRPM8 activation can inhibit the TAC1 expression and SP release by primary sensory neurons.

### TRPM8 activation may decrease SP release by inhibiting the Wnt/β-catenin signaling pathway in the ND7/23 cell line

The ND7/23 cell line, rat DRG/mouse N18Tg2 neuroblastoma hybridoma cell line, is commonly used for the study of sensory neuron function [18]. However, a previous study showed that *Tac1* was expressed at low levels in the ND7/23 cells [19]. Several studies have demonstrated that nerve growth factor (NGF) can promote the production of neuropeptides by inducing ND7/23 cells differentiation [20, 21]. Therefore, we differentiated ND7/23 cells using a neuronal culture medium that was used for DRG neuron culture, containing 50 ng/mL mouse NGF. The results showed that as the number of days of differentiation increased, the protein and mRNA levels of SP increased significantly and TRPM8 protein and mRNA levels decreased significantly (Supplementary Fig. 5A–C). In addition, our study found that the ratio of phosphorylated P38 to total P38 increased (Supplementary Fig. 5D, E), ratio of phosphorylated ERK1/2 to total ERK1/2 decreased, total ERK1/2 increased (Supplementary Fig. 5F, G), and total β-catenin increased (Supplementary Fig. 5H, I) with an increasing number of differentiation days.

**Fig. 3:**
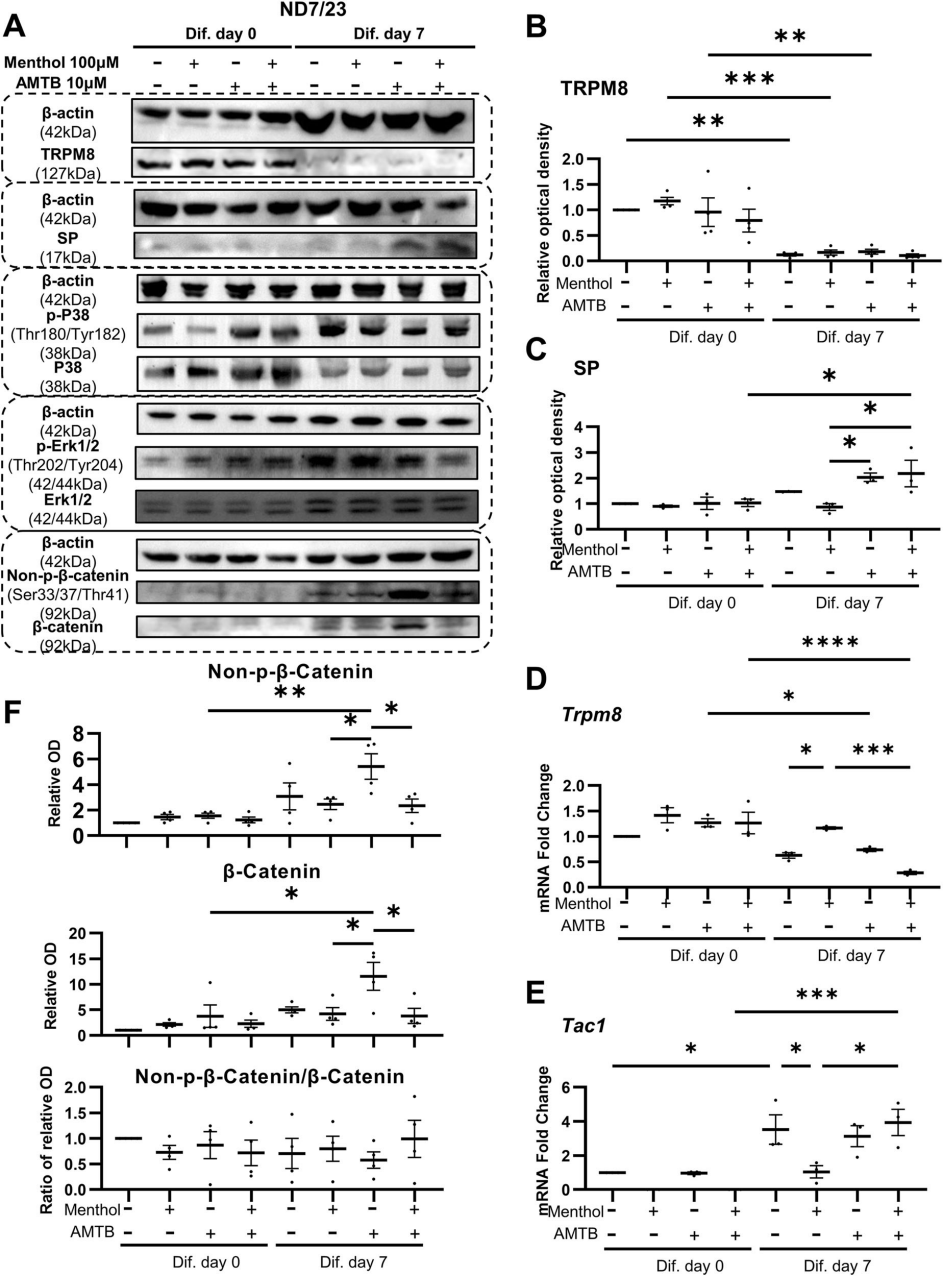
TRPM8 inhibit SP release by inhibiting Wnt/β-catenin signaling pathway in ND7/23 cells. **A** With or without the application of 100 μM menthol or/and 10 μM AMTB before and after differentiation in ND7/23 cells, the protein levels of TRPM8, SP, phosphorylated P38 and total P38, phosphorylated ERK1/2 and total ERK1/2, non-phosphorylated β-catenin and total β-catenin were detected by immunoblot. The relative optical densities of TRPM8 (**B** n = 4) and SP (**C**, n = 3) were calculated. **D**, **E** The mRNA fold changes of *Trpm8* and *Tac1* in ND7/23 cells with or without the application of menthol or/and AMTB were calculated (n = 3). **F** The relative optical densities of non-phosphorylated β-catenin and total β-catenin and ratio of non-phosphorylated β-catenin to total β-catenin (n = 4) were calculated. *P < 0.05; **P < 0.01; ***P < 0.001; ****P < 0.0001.

To clarify which signaling pathway plays the major role, we further stimulated the differentiated ND7/23 cells with menthol and/or AMTB for 6 h on day 7. Our results showed that TRPM8 protein level and mRNA expression were significantly decreased after differentiation and mRNA expression increased in the menthol-stimulated group (Fig. 3A, B, D). In contrast, SP protein and mRNA expression levels increased after differentiation and significantly decreased in the menthol-stimulated group (Fig. 3A, C, E). Furthermore, no significant difference in the MAPK/ERK pathway was observed (Supplementary Fig. 6A, B), whereas the ratio of phosphorylated P38 to total P38 increased after differentiation and slightly decreased after menthol stimulation (Supplementary Fig. 6C, D). Furthermore, the total β-catenin in the differentiated AMTB-stimulated group increased more than ten-fold compared with the control group and increased more than two-fold compared with the differentiated control group and the menthol-stimulated group (Fig. 3A, F). Moreover, the trend of changes in non-phosphorylated β-catenin was similar to that of β-catenin, while the ratio of non-phosphorylated β-catenin to total β-catenin remained unchanged (Fig. 3A, F). Our study suggested that TRPM8 activation may inhibit SP production by reducing total β-catenin level in ND7/23 cells.

### Wnt/β-catenin signaling is upregulated in colitis

**Fig. 4:**
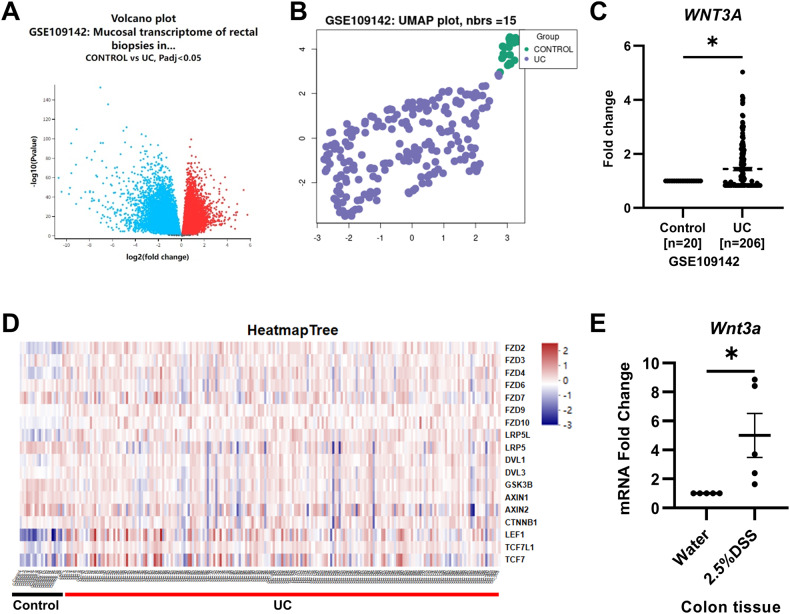
Wnt/β-catenin signaling was upregulated in colitis. **A** Volcano plot and **B** UMAP plot of mRNA expression differences in GSE109142 dataset were generated by GEO2R. **C** mRNA fold change of *WNT3A* in normal tissues (n = 20) and UC tissues (n = 206) from GSE109142. **D** Heatmap of expression of FZD2, FZD3, FZD4, FZD6, FZD7, FZD9, FZD10, LRP5L, LRP5, DVL1, DVL3, GSK3B, AXIN1, AXIN2, CTNNB1, LEF1, TCF7L1, and TCF7 in normal tissues (n = 20) and UC tissues (n = 206) from GSE109142. **E** *Wnt3a* was overexpressed in the colon tissues of the DSS-induced colitis group (n = 8). *P < 0.05.

Recent study has found that the activation of Wnt/β-catenin signaling pathway in inflammatory bowel disease can promote the expression of pro-inflammatory factors in Treg cells [22]. Earlier study analyzed the expression of WNT ligands and its receptor Frizzled (FZD) in UC patients and found that the expression of WNT ligands was generally increased in UC patients, with the most significant difference in WNT3A expression [23]. In addition, the expression of FZD was also significantly increased in UC patients [23]. To clarify Wnt/β-catenin signaling expression in patients with UC, we analyzed the high-throughput RNA sequencing data (GSE109142) from the Predicting Response to Standardized Pediatric Colitis Therapy (PROTECT) study, which included 20 controls and 206 UC patients [24]. The analysis results showed that the gene expression in the colon tissue of the control group and UC patients was significantly different (Fig. 4A, B), and the expression of *WNT3A* was increased in UC patients (Fig. 4C). In addition, the analysis also found that the expression of downstream molecules of the Wnt/β-catenin signaling such as *FZD2*, *FZD4*, *DVL1*, *CTNNB1*, *LEF1*, and *TCF7* was increased in UC patients, while the expression of inhibitory molecules *GSK3B* and *AXIN1/2* was decreased in UC patients (Fig. 4D). Furthermore, our results showed that *Wnt3a* were significantly upregulated in the DSS-induced colitis group (Fig. 4E).

### TRPM8 inhibits Wnt/β-catenin signaling via PKA/GSK-3β interaction

**Fig. 5:**
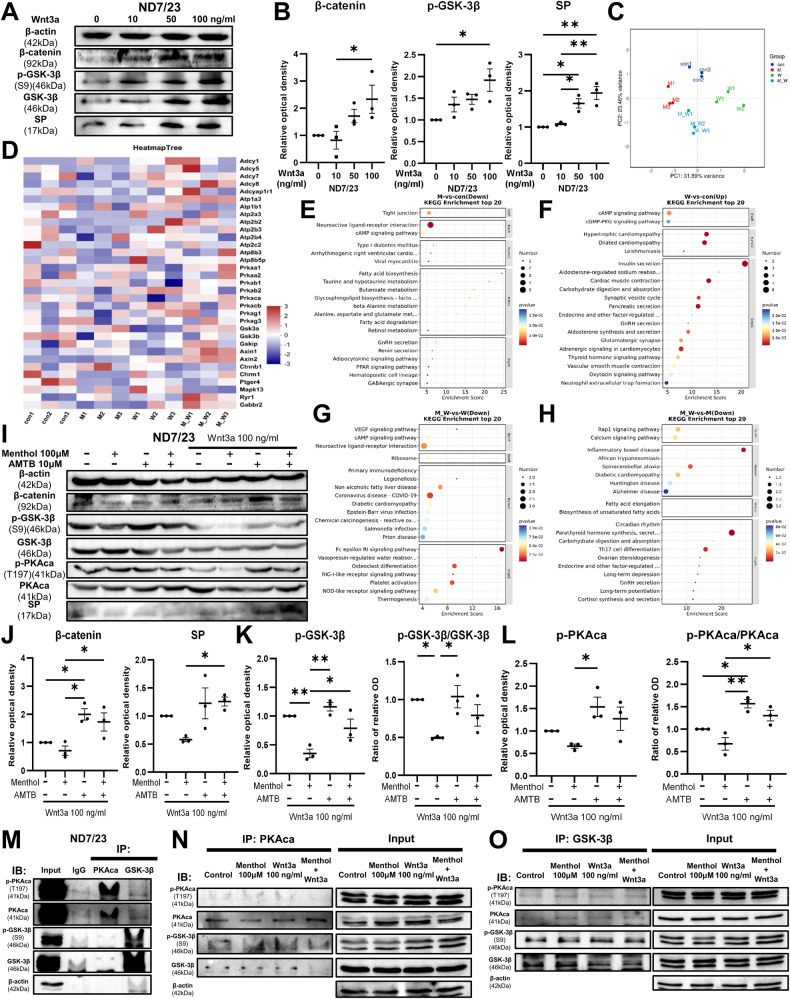
TRPM8 inhibits Wnt/β-catenin signaling-induced SP release via PKA/GSK-3β interaction in ND7/23 cells. **A** The protein levels of β-catenin, phosphorylated GSK-3β, total GSK-3β and SP in ND7/23 cells after stimulation of Wnt3a for 6 h, and β-actin was used as a loading control. **B** The relative optical densities of β-catenin, phosphorylated GSK-3β and SP in ND7/23 cells after stimulated of Wnt3a (n = 3). We stimulated ND7/23 cells with 100 μM menthol, 100 ng/ml Wnt3a and menthol with Wnt3a for 6 h and performed RNA sequencing analysis. **C** A PCA plot was generated to represent the genes expression trends in the samples. **D** A heatmap of gene expression related to cAMP signaling pathway and Wnt/β-catenin signaling pathway was generated. Top 20 significant enrichments of KEGG pathways were presented to compare control vs menthol (**E**), control vs Wnt3a (**F**), menthol with Wnt3a vs Wnt3a (**G**), and menthol with Wnt3a vs menthol (**H**). **I** The protein levels of β-catenin, phosphorylated GSK-3β, total GSK-3β, phosphorylated PKAca, total PKAca and SP in ND7/23 cells after stimulation of 100 μM menthol or/and 10 μM AMTB or/and 100 ng/ml Wnt3a for 6 h. **J** The relative optical densities of β-catenin and SP in Wnt3a stimulated groups (n = 3). The relative optical densities and ratio of phosphorylated GSK-3β and total GSK-3β (**K**), phosphorylated PKAca and total PKAca (**L**) in Wnt3a stimulated groups (n = 3). **M** immunoblot analysis of the indicated proteins from immunoprecipitates (IP) via PKAca antibody, GSK-3β antibody, and IgG, and input group obtained from ND7/23 cells. immunoblot analysis of the indicated proteins from IP via PKAca antibody (**N**) and GSK-3β antibody (**O**) and input groups from ND7/23 cells that stimulated by 100 μM menthol, 100 ng/ml Wnt3a and menthol with Wnt3a for 6 h. The cell lysates were pulled down with nickel beads and immunoblotted with indicated antibody. *P < 0.05; **P < 0.01.

To verify the effect of Wnt3a on the ND7/23 cell line, we stimulated the cells with gradient concentrations of Wnt3a and performed immunoblot. The results showed that Wnt3a significantly increased the level of β-catenin, phosphorylated GSK-3β and SP (Fig. 5A, B), but had no statistical significance on the level of GSK-3β (Supplementary Fig. 7A). In addition, Wnt3a downregulated the *Trpm8* expression in ND7/23 cell line (Supplementary Fig. 7B). To clarify how TRPM8 inhibits the Wnt/β-catenin signaling, we stimulated ND7/23 cells with 100 μM menthol or/and 100 ng/ml Wnt3a for 6 h and performed RNA sequencing. The results showed that the different stimuli can dramatically affect gene expression in cells (Fig. 5C). Genes related to adenylate cyclase and sodium-potassium-ATPase were upregulated in Wnt3a group and downregulated in menthol group (Fig. 5D). Through KEGG enrichment analysis, the application of menthol inhibited the cAMP signaling (Fig. 5E), and the application of Wnt3a promoted the cAMP signaling (Fig. 5F), while menthol could inhibit the promoting effect of Wnt3a on the cAMP signaling (Fig. 5G), and Wnt3a could inhibit the calcium signaling induced by menthol (Fig. 5H).

GSK-3β is the main inhibitory molecule in Wnt/β-catenin signaling pathway [25]. PKA is the main molecule of the cAMP signaling pathway [26], among which PKAca is the main catalytic subunit of PKA [27]. We further verified their protein levels under the activation of TRPM8 and/or Wnt3a. The results showed that menthol inhibited the promotion effect of Wnt3a on the protein levels β-catenin and SP (Fig. 5I, J). Under Wnt3a stimulation, menthol decreased the level of phosphorylated GSK-3β and the ratio of phosphorylated GSK-3β to total GSK-3β (Fig. 5K), and had the same inhibitory effect on PKAca (Fig. 5L). In the absence of Wnt3a stimulation, the levels of various proteins were not statistically different among the four groups (Supplementary Fig. 7C–F). The above results proved that there was a clear positive correlation between the phosphorylation degree of GSK-3β and the phosphorylation degree of PKAca on the basis of the activation of Wnt/β-catenin signaling pathway.

In order to clarify whether there is an interaction between PKAca and GSK-3β, we used PKAca and GSK-3β antibodies to immunoprecipitate ND7/23 cell lysates and performed immunoblot. We found that phosphorylated GSK-3β protein was present in the immunoprecipitation group using PKAca antibody, and phosphorylated PKAca protein was also present in the immunoprecipitation group using GSK-3β antibody (Fig. 5M). We next performed immunoprecipitation and immunoblot on menthol and/or Wnt3a-stimulated ND7/23 cells. The results showed that all four groups had the combination of PKAca and GSK-3β, and in the PKAca and GSK-3β immunoprecipitation groups, menthol inhibited the combination of PKAca and GSK-3β under the action of Wnt3a (Fig. 5N, O). In addition, menthol decreased the level of phosphorylated GSK-3β in the corresponding input group (Fig. 5N, O). The above results demonstrated that phosphorylated PKAca and GSK-3β can be combined with each other, which explains the positive correlation phenomenon of their phosphorylation degree.

### SP inhibits proliferation and promotes apoptosis of colonic organoids

**Fig. 6:**
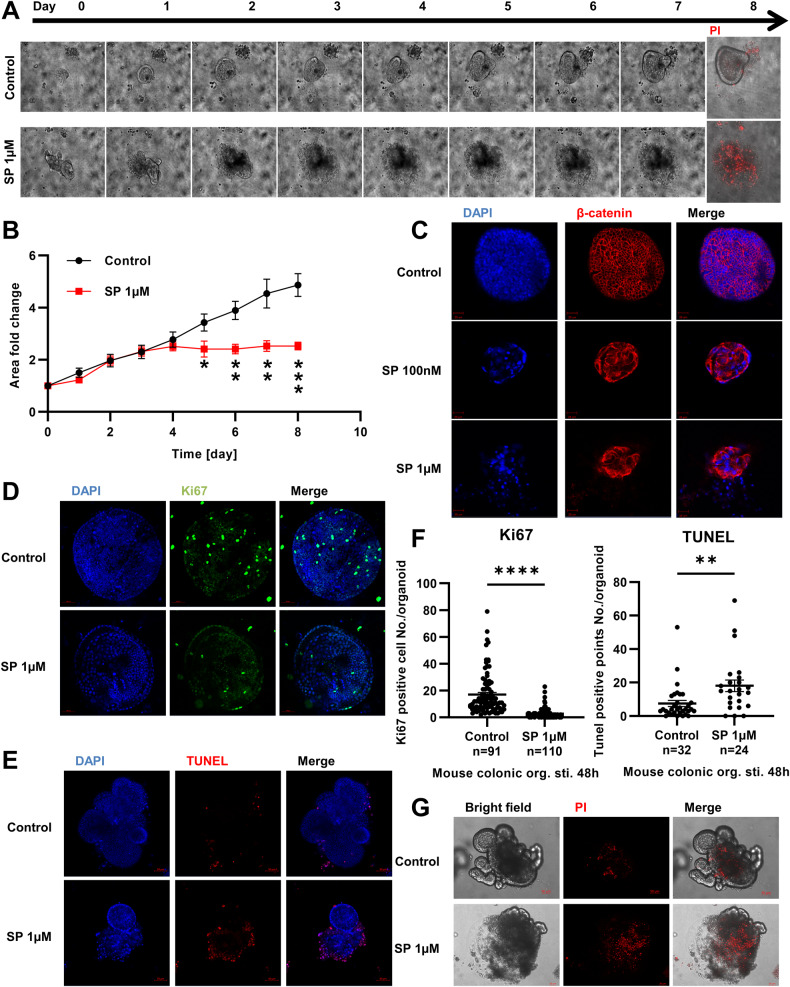
SP regulates the proliferation and apoptosis in mouse colonic organoids. **A** Mouse colonic organoids were stimulated with SP (1 μM) for 8 days and the same organoids were performed microscopy every day, and stained with PI (red) on day 8. **B** The area of the organoids was measured every day to draw a fold change curve (n = 6). **C** Immunostaining of organoids stimulated with different concentrations of SP (100 nM or 1 μM) for 48 h, DAPI (blue) indicates cell nuclei, and β-catenin (red) indicates cell outlines (living cells). After SP stimulation for 48 h, Ki67, TUNEL and PI staining was performed in colonic organoids, Ki67 (green) indicated proliferating cells (**D**), and TUNEL or PI (red) indicated apoptosis (**E**, **G**). **F** The Ki67-positive cells or TUNEL-positive particles of each organoid were counted to compare the effect of SP on proliferation and apoptosis, n indicated the number of organoids. *P < 0.05; **P < 0.01; ***P < 0.001; ****P < 0.0001.

The nerve endings of primary sensory neurons are distributed in the submucosa of the colon, and they secrete neuropeptides such as SP [28]. The SP receptor NK-1R is highly expressed in the colonic epithelium and is upregulated in IBD patients [14]. To observe the effect of SP on the colonic epithelium, we stimulated mouse colonic epithelial organoids with SP (1 μM) for 8 days and performed propidium iodide (PI) staining to indicate apoptosis. Our study found that SP inhibited the growth of colonic epithelial organoids and promoted their apoptosis (Fig. 6A). The area fold-change curves indicated that the organoids stopped growing after day 3, and the difference was significant (Fig. 6B). Similar effects were observed by stimulating colonic organoids with different concentrations of SP (100 nM and 1 μM) for 48 h (Fig. 6C). To clarify the role of SP in the proliferation and apoptosis of mouse colonic epithelial organoids, we performed Ki67, TUNEL, and PI staining. The results showed that SP stimulation decreased the number of Ki67-positive cells (Fig. 6D) and increased the number of TUNEL-positive particles (Fig. 6E), with a statistically significant difference (Fig. 6F). The PI staining images showed that SP significantly promoted apoptosis in mouse colonic organoids (Fig. 6G).

**Fig. 7:**
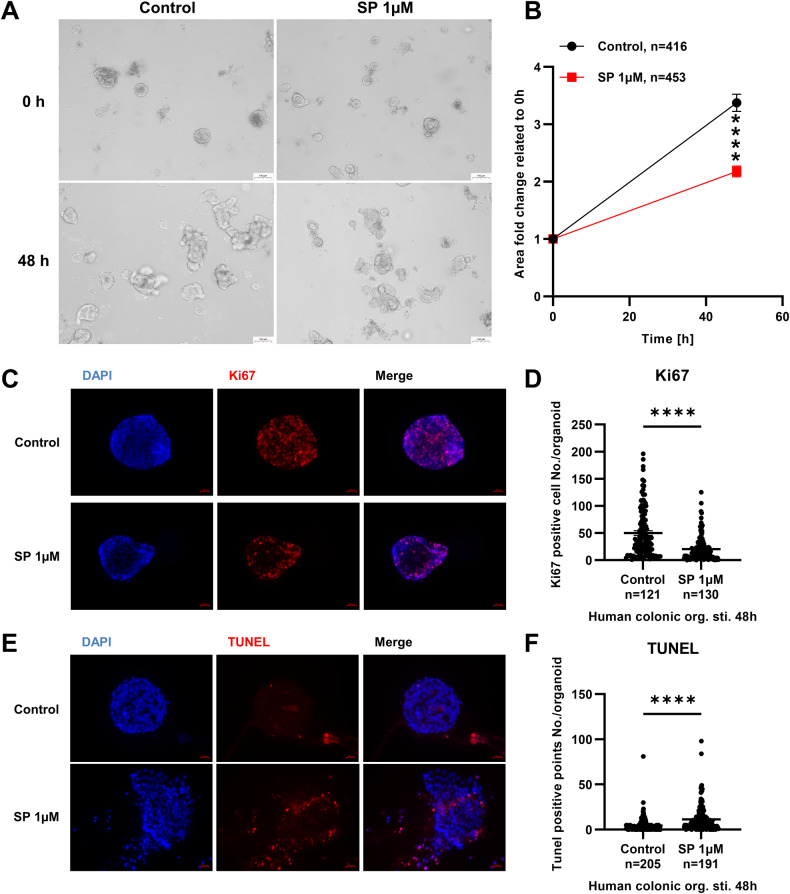
SP inhibits the proliferation and promote the apoptosis of human colonic organoids. **A** Human colonic organoids were stimulated by SP (1 μM) for 48 h and performed microscopy before and after stimulation. **B** The area of the organoids was measured before and after stimulation to draw a fold change curve, n indicated the number of organoids. **C** Ki67 staining of organoids stimulated by SP for 48 h, DAPI (blue) indicates cell nuclei and Ki67 (green) indicated proliferating cells. **D** The Ki67-positive cells of each organoid were counted; n indicated the number of organoids. **E** TUNEL staining of organoids after stimulation, DAPI (blue) indicates cell nuclei and TUNEL (red) indicated apoptosis. **F** The TUNEL-positive particles of each organoid were counted; n indicated the number of organoids. ****P < 0.0001.

To verify the effect of SP on human colonic epithelium, we stimulated human colonic organoids with SP (1 μM) for 48 h and performed microscopy and staining. The results showed that SP inhibited the growth of human colonic organoids (Fig. 7A), and organoids in the SP-stimulated group were significantly smaller than those in the control group (Fig. 7B). On the other hand, the staining showed that the number of Ki67-positive cells in human colonic organoids significantly decreased after SP stimulation (Fig. 7C, D), with smaller organoids (Fig. 7C). In contrast, the number of TUNEL-positive particles in SP-stimulated organoids were increased (Fig. 7E, F), and the 3-dimensional structure of the organoids was destroyed (Fig. 7E). Collectively, these results indicated that SP may aggravate IBD or DSS-induced colitis in mice by inhibiting proliferation and promoting apoptosis of colonic epithelial cells.

### The SP receptor antagonist Aprepitant and menthol can both alleviate colitis in mice but the effects are not additive

**Fig. 8:**
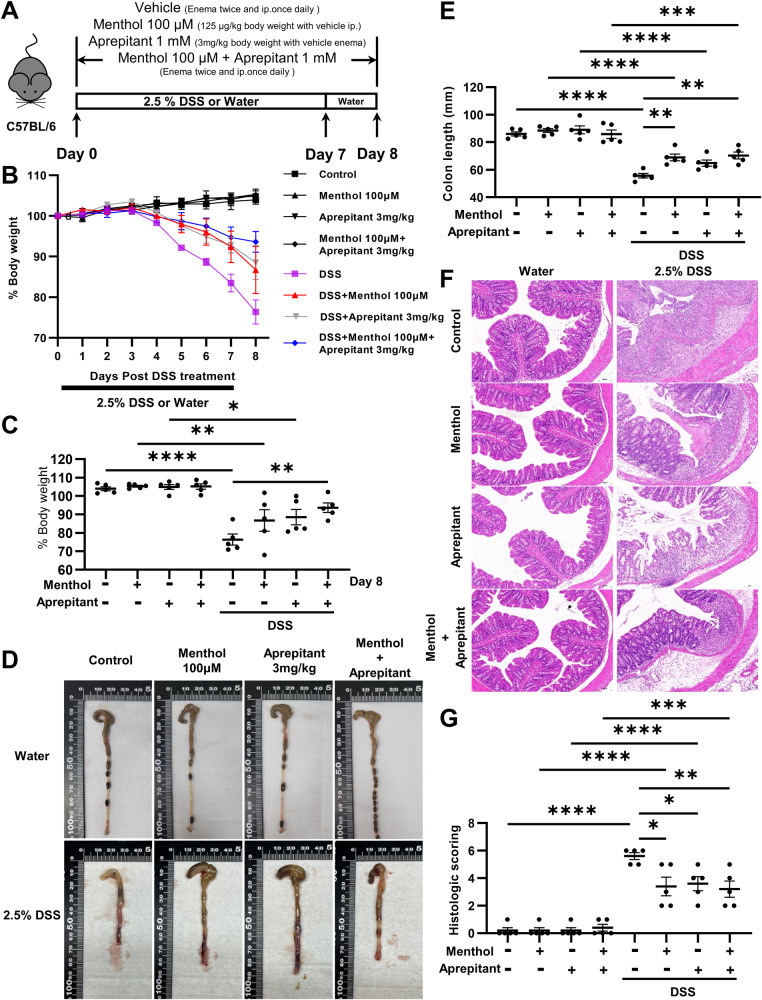
The SP receptor antagonist Aprepitant cannot enhance the therapeutic effect of menthol in DSS-induced mice colitis. **A** Grouping method, drug dosages and examination time points of animal experiments. **B** The body weight curves were drawn for eight groups of colitis and non-colitis mice (n = 5). **C** Comparison of body weight percentages on day 8 of eight groups of colitis and non-colitis mice (n = 5). **D** The photographs of colons with scale bars in eight groups of mice and **E** the summary graph (n = 5). **F** Colonic H&E staining pictures of eight groups mice and **G** the histological scoring graph (n = 5). *P < 0.05; **P < 0.01; ***P < 0.001; ****P < 0.0001.

Presently, SP receptor NK-1R antagonist (Aprepitant) has been utilized in the treatment of chemotherapy-induced nausea and vomiting [15], and could be used to relieve cough in lung cancer [29]. If the effect of Aprepitant in colitis is determined, Aprepitant could be regarded as a potential drug for IBD treatment. In addition, comparing the therapeutic effects of menthol, Aprepitant, and Aprepitant plus menthol in colitis can clarify whether they have a direct relationship. In the drinking water negative control groups or during DSS-induced colitis modeling in mice, menthol was given by enema and Aprepitant was given by intraperitoneal injection (Fig. 8A). Compared with drug application groups and negative control groups, the DSS group had more severe colitis symptoms, significantly reduced body weight (Fig. 8B, C), and significantly shortened colon (Fig. 8D, E), while there was no significant difference between the negative control groups (Fig. 8B–E). In addition, histopathological analyses of colonic tissues using H&E staining revealed significant infiltration of inflammatory cells and destruction of mucosal epithelial layer in the DSS group (Fig. 8F, G). However, there were no significant differences in body weight, colon length, inflammatory cell infiltration, and epithelial disruption among the drug-applied DSS groups (Fig. 8B–G). Experiments shown that both TRPM8 agonist menthol and SP receptor antagonist Aprepitant could alleviate colitis in mice, but they could not be superimposed, which verifies the role of TRPM8 in attenuating colitis by inhibiting SP release.

## Discussion

The sensory nervous system plays important role in IBD pathogenesis. TRP channels are nociceptors and temperature receptors in primary sensory neurons that regulate the secretion of neuropeptides [30]. TRPM8 is a cold-sensory receptor, and its specific agonist, menthol, is a widely used food additive and is found in traditional Chinese medicine [31–33]. Previous study reported that the activation of TRPM8 inhibited the release of the pro-inflammatory factor TNF-α in macrophages through the MAPK pathway and promoted the release of the anti-inflammatory factor, IL-10 [10]. Our study identified a different mechanism whereby TRPM8 activation can inhibit the secretion of the pro-inflammatory neuropeptide SP from the colonic primary sensory neurons [34].

TRPM8 induces calcium ion influx in primary sensory neurons and affects various cellular functions. In macrophages, TRPM8 regulates MAPK signaling pathways such as MAPK/ERK and MAPK/P38 [10]. Another study showed that TRPM8 activation can inhibit the development of colorectal cancer by suppressing the Wnt/β-catenin signaling pathway [35]. Our study discovered for the first time that the activation of TRPM8 mainly inhibited the SP release from primary sensory neurons via Wnt/β-catenin signaling pathway. In addition, we found that Wnt3a, a ligand of the Wnt/β-catenin signaling pathway, was upregulated in the lesion tissues of IBD patients and colitis mice, and TRPM8 can inhibit the combination of PKAca molecule of cAMP signaling pathway and GSK-3β molecule of Wnt/β-catenin signaling pathway, thereby inhibiting GSK-3β phosphorylation. GSK-3β is the main inhibitory molecule of Wnt/β-catenin signaling pathway, which is inactivated by degradation after phosphorylation [25]. Unphosphorylated GSK-3β can phosphorylate β-catenin, thereby promoting the degradation of β-catenin and inhibiting its translocation into the nucleus to regulate transcription [25].

Colonic epithelial barrier function is impaired in IBD and the SP receptor NK-1R is expressed in the epithelial cells [36, 37]. Several studies have shown that short-term (6–24 h) stimulation of SP can inhibit apoptosis in the colonic epithelial cell line NCM460 [38, 39]. Another study found that long-term (48 h) stimulation of SP can induce apoptosis in B16F10 melanoma cells [40]. Similarly, our study revealed that SP stimulation for less than 24 h had no significant effect on the growth of mouse colonic organoids, whereas SP stimulation for 48 h inhibited proliferation and promoted apoptosis of mouse and human colonic organoids. Our results indicated that the effect of SP on colonic epithelium was time dependent. IBD is a chronic inflammatory disease, the long-term stimulation of SP may be closer to the pathogenesis of IBD, and SP may aggravate the severity of IBD by promoting colonic epithelial cell apoptosis. SP receptor NK-1R antagonist (Aprepitant) has been used clinically to treat nausea, vomiting and cough [15, 29]. Our study found that menthol and Aprepitant have clear therapeutic effects on DSS-induced colitis in mice, and they can be regard as potential therapeutic drugs for IBD. These two substances have no obvious toxic and side effects on the human body, and further relevant clinical trials should be carried out. Our research mainly focused on mouse model experiments, so the regulatory effect of TRPM8 on SP release in colonic tissues of patients with IBD should be further studied.

Collectively, our study showed that the expression levels of *TRPM8*, *TAC1*, *WNT3A* were correlated with the severity of IBD. The activation of TRPM8 attenuated DSS-induced colitis in mice. Menthol-induced calcium influx of TRPM8 can inhibit the combination of PKAca from the cAMP signaling pathway and GSK-3β from the Wnt/β-catenin signaling pathway, thereby inhibiting the role of β-catenin in promoting the release of SP from primary sensory neurons. Our study also found that long-term SP stimulation inhibited proliferation and promoted apoptosis in mouse and human colonic epithelial organoids. Both the TRPM8 agonist menthol and the SP receptor antagonist Aprepitant can attenuate colitis in mice, but the effects were not additive.

## Materials and methods

### Experimental animals

8–12 weeks old C57BL/6 mice were purchased from Model Organisms Center. All animal experiments were approved by the Biological Research Ethics Committee of the Tongji University (Permit Number: TJBB05523101). Mice were administered 2.5% DSS (MP Biomedicals) to induce colitis [10]. Menthol was dissolved and stored in DMSO at 100 mM and administrated as 125 μg/kg body weight [10]. Aprepitant was performed as 3 mg/kg body weight [41].

### Histological analysis of colitis

Colitis severity was determined by assessing the sum (range, 0–6) of the scores (range, 0–3) for the degree of tissue damage and lamina propria inflammatory cell infiltration [42].

### Dorsal root ganglion isolation and stimulation

The mice dorsal root ganglion (DRG) was dissected and dissociated, as previously described [43, 44].

### Ratiometric (Ca^2+^)i measurements

Ratiometric (Ca^2+^)i measurements were performed using a Fluo-4 Calcium Imaging Kit (Thermo Fisher, New York, USA) [45].

### Colonic crypt isolation and organoid culture

The specific experimental procedure has been described previously and the details was in the online supplementary methods [43].

### Substance P release from isolated mouse colon

The details of the experimental procedure were performed as Engel M.A.’s previous study and see online supplementary methods for details [16, 46, 47].

### Statistical analysis

All results are presented as the mean ± standard error of the mean (SEM). The number (n) quoted throughout the manuscript refers to the number of mice, isolated DRG neurons, colonic organoids, or experimental repetitions. The t-test was used to compare two groups, and one-way ANOVA and Tukey’s multiple comparisons test were used to compare more than two groups to determine the statistical significance. All results were calculated using GraphPad Prism V9: *P < 0.05, **P < 0.01, ***P < 0.001, and ****P < 0.0001.

### Supplementary information

Supplemental Materials

aj-checklist

## Data Availability

All data needed to evaluate the conclusions in the paper are present in the paper or the Supplementary Materials. The RNA-sequencing data presented in this article have been submitted to the NCBI Gene Expression Omnibus database under accession number GSE242559.
