# Supplementary material for: TRPM8 inhibits substance P release from primary sensory neurons via PKA/GSK-3beta to protect colonic epithelium in colitis
Source: Cell Death Dis. 2024 Jan 27;15(1):91. doi: 10.1038/s41419-024-06480-5 (PMC10821925; doi:10.1038/s41419-024-06480-5)
Supplement: Supplementary file 1 — Supplemental Materials [file 41419_2024_6480_MOESM1_ESM.pdf]

Supplementary Figures

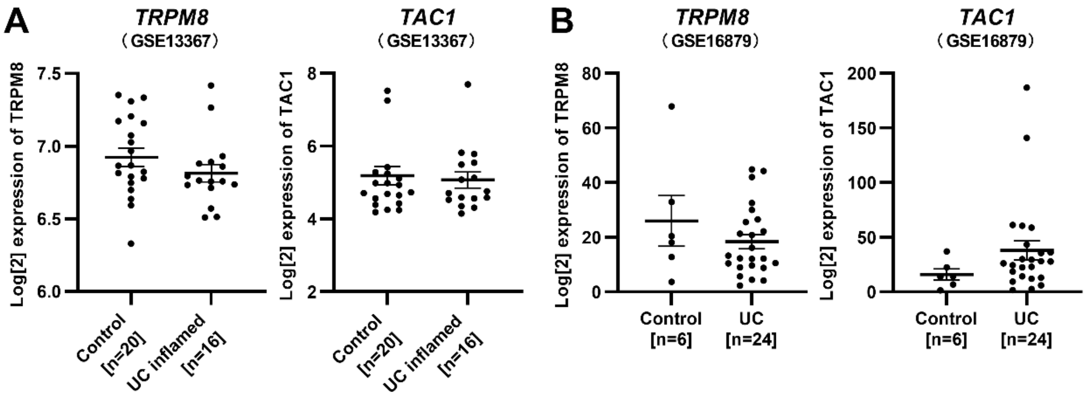

Supplementary Fig. 1 The expression of TRPM8 and TAC1 in GSE13367 and GSE16879. (A) The mRNA expression of *TRPM8* and *TAC1* in control tissues (n=20) and UC inflamed tissues (n=16) indicated by data from GSE13367. (B) The mRNA expression of *TRPM8* and *TAC1* in normal tissues (n=6) and UC tissues (n=24).

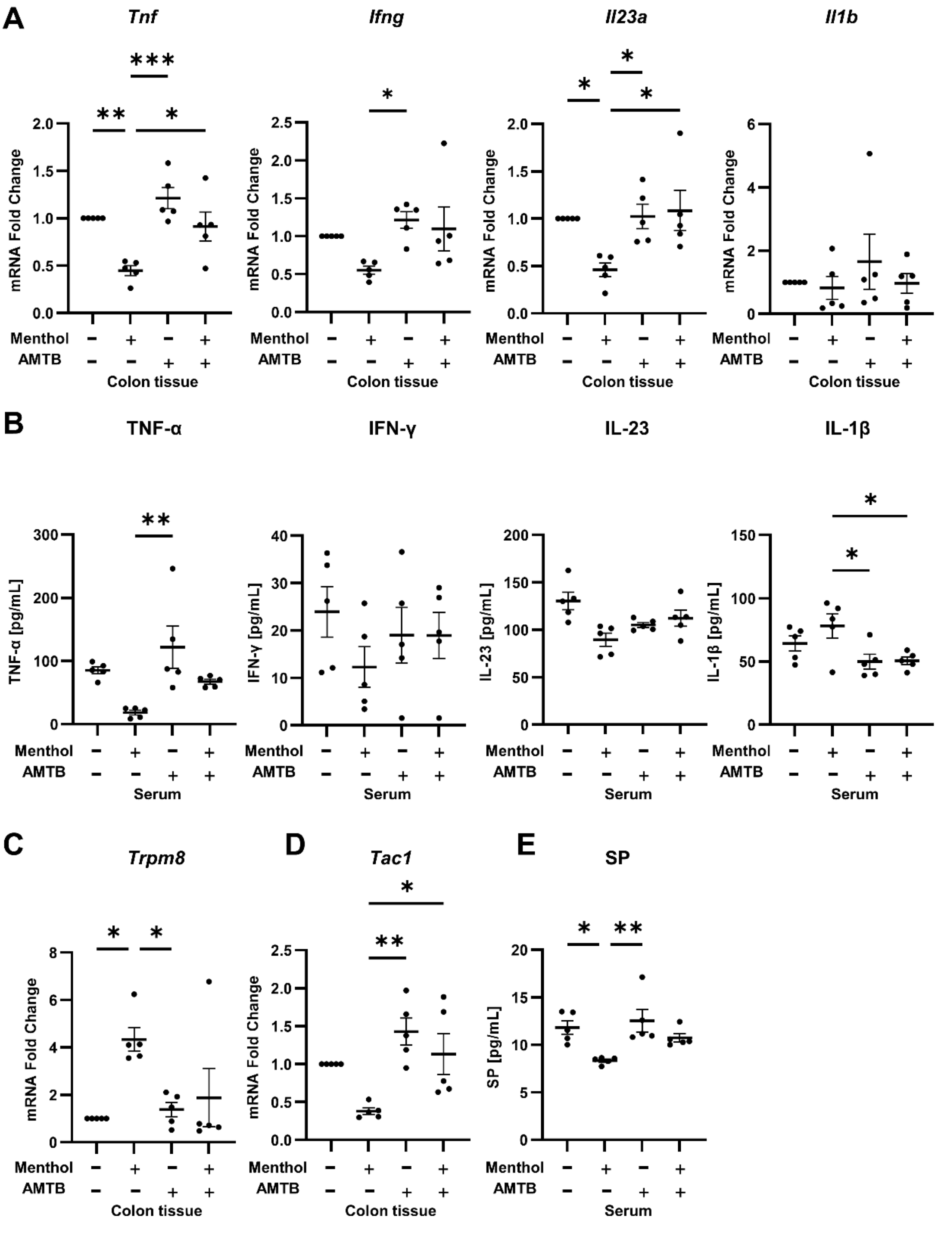

Supplementary Fig. 2 Menthol regulates the inflammatory cytokines and SP release in colitis mice. (A) The mRNA fold changes of *Tnf, Ifng, Il23a,* and *Il1b* in the four groups (n=5). (B) The sera of the four groups mice were measured for TNF-α, IFN-γ, IL-23, and IL-1β by ELISA (n=5). (C) The mRNA expression fold change of *Trpm8* in the four groups (n=5). (D) The mRNA expression fold change of *Tac1* and (E) the

protein level of SP in the four groups of colitis mice (n=5). *P < 0.05; **P < 0.01; ***P < 0.001.

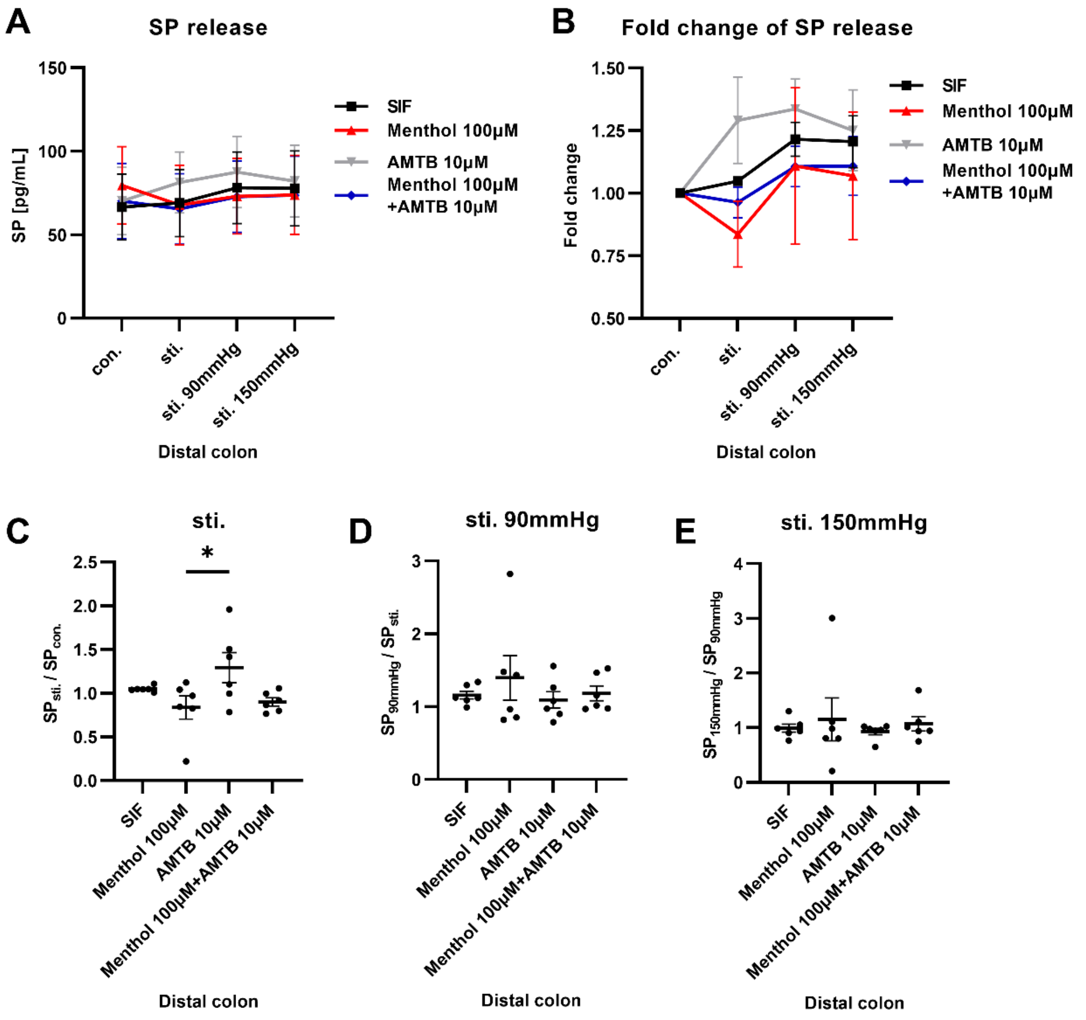

Supplementary Fig. 3 The activation of TRPM8 inhibits SP release from mouse colon and is reversed by nociceptive stimuli. (A) The concentrations of SP released by the mouse colon under different pressurized conditions (no pressure or 90 mmHg or 150 mmHg) and different stimulation (menthol 100μM or/and AMTB 10μM) was determined by ELISA, n=6. (B) The concentrations of SP released by the mouse

colon were based on the control stage to make a fold change curve. In order to clarify the difference between the different stages among the groups, (C) we compared the ratio of stimulation stage and control stage, (D) the ratio of 90 mmHg stage and stimulation stage, (E) the ratio of 150 mmHg stage and 90 mmHg stage. *P < 0.05.

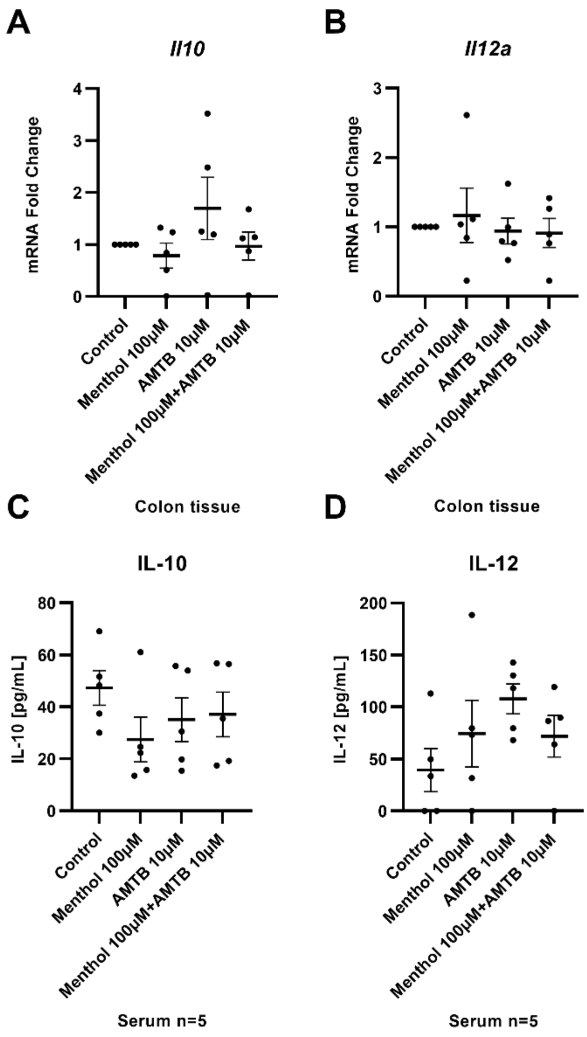

Supplementary Fig. 4 Menthol affects the inflammatory cytokines release in colitis mice. (A, B) The mRNA fold changes of *Il10* and *Il12a* in the four groups (n=5). (C, D) The sera of the four groups mice were measured for IL-10 and IL-12 by ELISA (n=5).

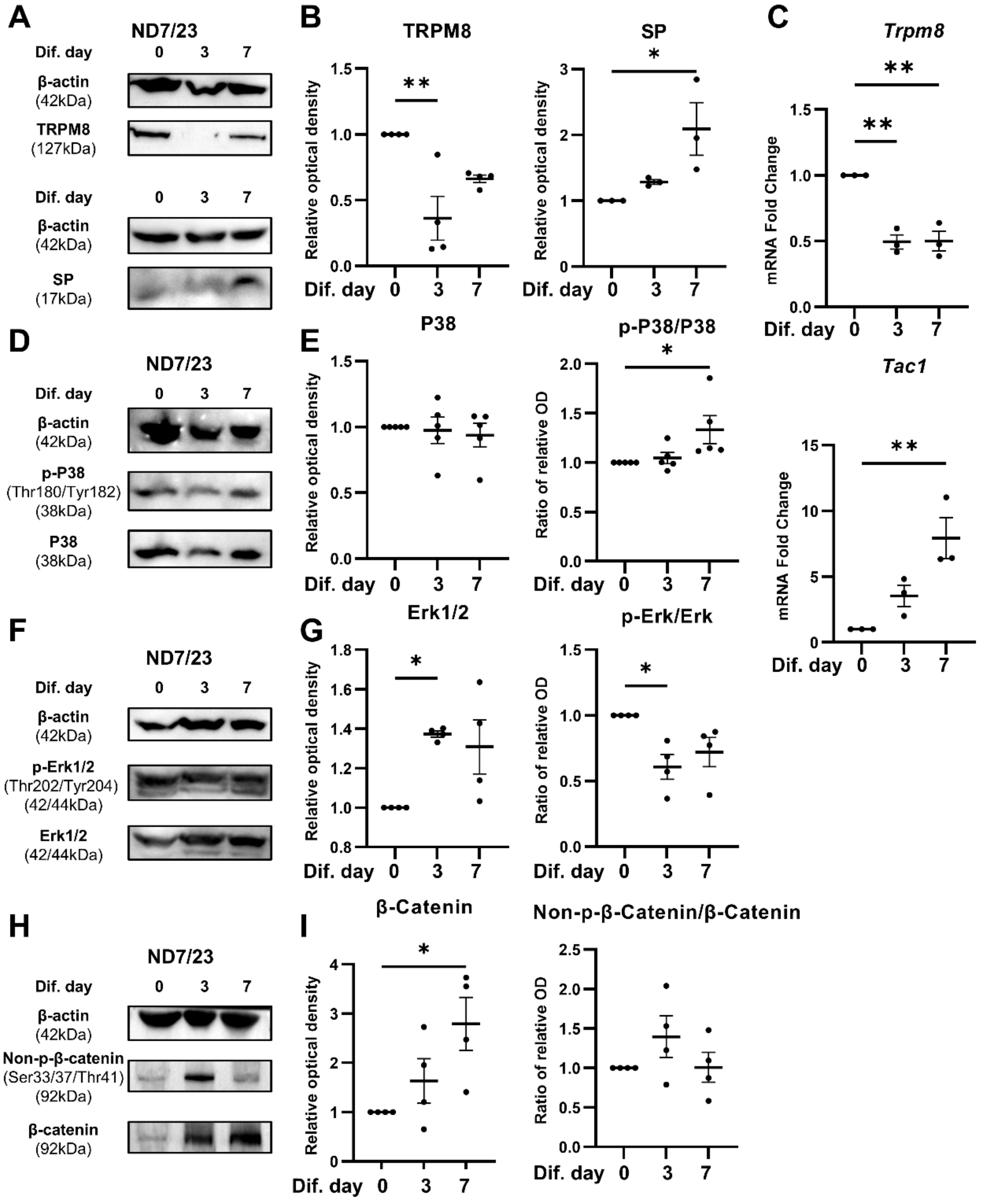

Supplementary Fig. 5 The differentiation of ND7/23 cells affects the protein levels. (A) Immunoblotting was used to detected the protein levels of TRPM8 and TAC1 in ND7/23 cells after 3 days and 7 days of differentiation, and β-actin was used as a loading control. (B) The relative optical densities of TRPM8 and SP in ND7/23 cells

after 3 days and 7 days of differentiation (n=4). (C) The mRNA fold changes of *Trpm8* and *Tac1* in ND7/23 cells after 3 days and 7 days of differentiation (n=3). The protein levels of phosphorylated P38 and total P38 (D), phosphorylated ERK1/2 and total ERK1/2 (F), non-phosphorylated β-catenin and total β-catenin (H) in ND7/23 cells after differentiation, and the relative optical densities or ratios corresponding to the above proteins (E n=5,G n=4,I n=4). *P < 0.05; **P < 0.01.

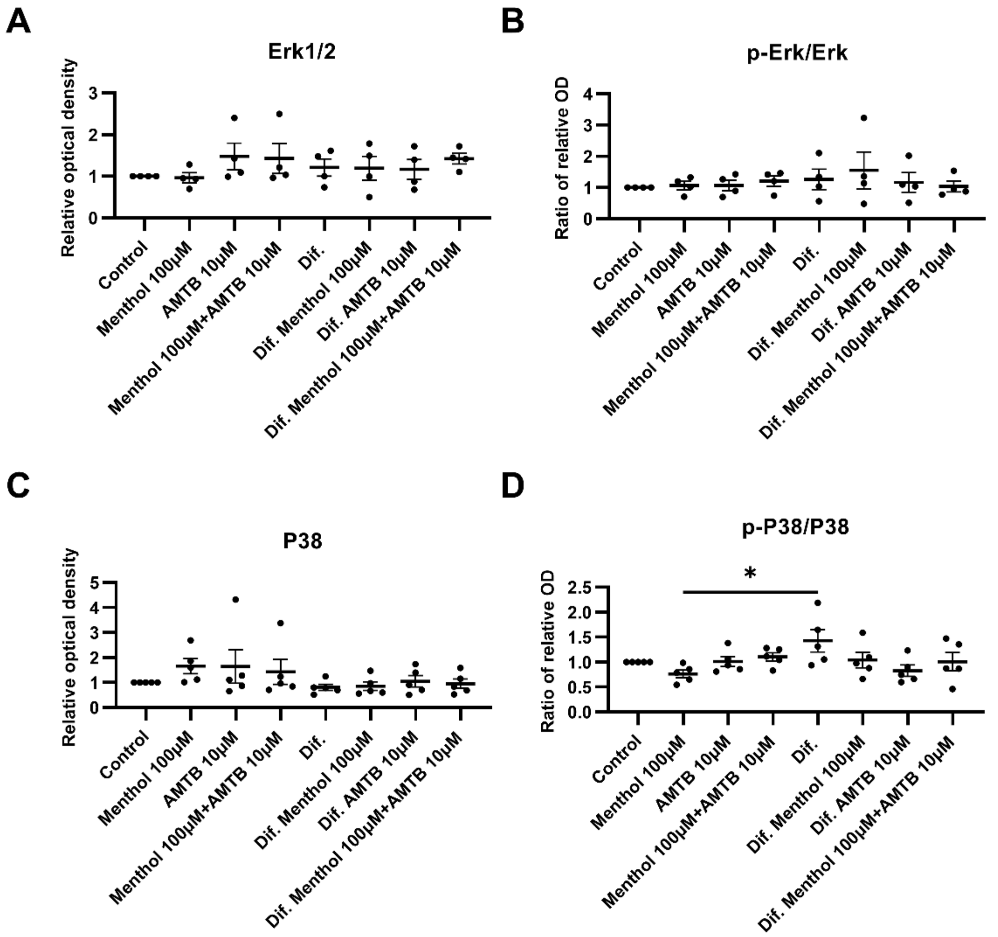

Supplementary Fig. 6 TRPM8 influences the protein level in undifferentiated and differentiated ND7/23 cells. The relative optical densities and ratios of

phosphorylated P38 to total P38 (A, B, n=4) and phosphorylated ERK1/2 to total ERK1/2 (C, D, n=5). *P < 0.05.

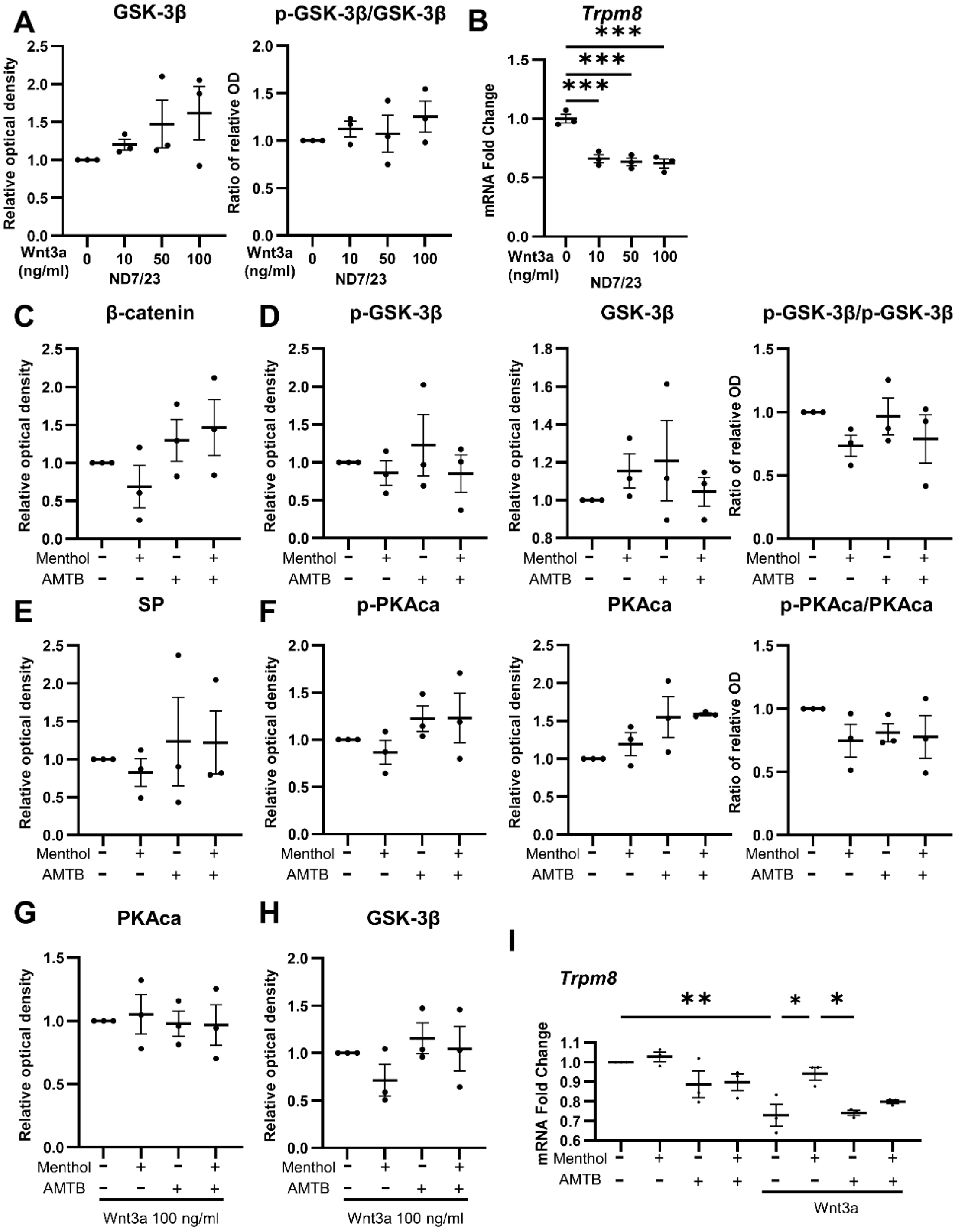

Supplementary Fig. 7 The effects of TRPM8 on PKAca and GSK-3β in ND7/23 cells. (A) The relative optical densities and ratio of phosphorylated GSK-3β and total GSK-3β in ND7/23 cells after stimulation of Wnt3a for 6 h (n=3). The mRNA fold changes

of *Trpm8* in ND7/23 cells after stimulated by different concentration of Wnt3a (B) or 100 μM menthol or/and 10 μM AMTB or/and 100 ng/ml Wnt3a (I) for 6 h (n=3). The relative optical densities of β-catenin (C) and SP (E) in non-Wnt3a stimulated groups (n=3). The relative optical densities and ratio of phosphorylated GSK-3β and total GSK-3β (D), phosphorylated PKAca and total PKAca (F) in non-Wnt3a stimulated groups (n=3). The relative optical densities of PKAca (G) and GSK-3β (H) in Wnt3a stimulated groups (n=3). *$P < 0.05$; **$P < 0.01$; ***$P < 0.001$.

Supplementary Methods

Experimental Animals

Sex-matched and 8–12 weeks old C57BL/6 mice (Model Organisms Center, Shanghai, China) were housed in a temperature- and light-controlled specific-pathogen-free room 12 h light and 12 h dark cycle with 55–60% humidity at 23 ± 1 °C and given standard sterile food and water. All animal maintenance and experimental procedures were performed in accordance with the Tongji University Guide for the Use of Laboratory Animals. Mice were randomly divided into cages (5 mice per cage) and administered oral 2.5% DSS (molecular weight: 36–50 kDa; MP Biomedicals, Illkirch, France) in drinking water for 7 days (the solution was changed every other day) to induce colitis. On the 7th day, the DSS solution was replaced with normal drinking water, and the experiment ended on the 8th day. For non-colitis and colitis experiment, non-colitis group was administered water, and colitis group was administered 2.5% DSS for 7 days, continue to drink normal water for 1 day. For drug application experiment, from day 0 to day 8, the mice were administered enemas twice or/and intraperitoneal injection once daily for 8 days under light isoflurane anesthesia (control group: phosphate buffered saline (PBS; without calcium and magnesium, Biosharp, Beijing, China), performed same volume as menthol group; agonist group: menthol, 100 μM (Merck KGaA, Darmstadt, Germany), 125 μg/kg body weight; antagonist group: AMTB, 10 μM (Merck KGaA, Darmstadt, Germany), performed same volume as menthol group; agonist + antagonist group: menthol 100 μM + AMTB 10 μM, performed same volume as menthol group) (menthol and AMTB

were dissolved and stored in DMSO at 100 mM and 10 mM respectively); Aprepitant group: Aprepitant, 1 mM (Abmole Bioscience Inc., Houston, USA), 3mg/kg body weight. The mice body weights were recorded daily and colonic endoscopy was performed at 0.5–1 cm from the anus on day 7 under light isoflurane anesthesia. Blood was obtained from the posterior ophthalmic artery of the mice on day 8 and serum was obtained by coagulation and centrifugation (4000 rpm, 4°C, 10 min). After the mice were sacrificed on day 8, the entire colon length was measured and the distal colon tissue was retained. The record-keeper was unaware of the grouping.

Histological analysis of colitis

The excised distal colons of the mice were fixed with 4% paraformaldehyde (PFA; Biosharp, Beijing, China) for 24 h and then embedded with paraffin. The paraffin cross-sections (5 mm) were stained with hematoxylin and eosin (H&E). Colitis severity was determined by assessing the sum (range, 0–6) of the scores (range, 0–3) for the degree of tissue damage and lamina propria inflammatory cell infiltration. Tissue damage scoring was as follows: 0, ¼ normal; 1, ¼ isolated focal epithelial damage; 2, ¼ mucosal erosions and ulcerations; and 3, ¼ extensive damage deep into the bowel wall. Lamina propria inflammatory cell infiltration scoring was as follows: 0, ¼ infrequent; 1, ¼ increased, some neutrophils; 2, ¼ submucosal presence of inflammatory cell clusters; and 3, ¼ transmural cell infiltrations.

Dorsal root ganglion isolation and stimulation

The mice dorsal root ganglion (DRG) was dissected and dissociated. After dissociation, the DRG neurons were resuspended in 5 ml neuronal culture medium

(neurobasal medium; Thermo Fisher, New York, USA) with B-27 supplement (Thermo Fisher), penicillin/streptomycin (100 units/ml and 100 μg/ml, Thermo Fisher), L-glutamine (2 mM, Thermo Fisher), mouse NGF (50 ng/mL, Thermo Fisher), and cytosine arabinoside (10 μM, Sigma, Steinheim, Germany). After centrifugation (400 g, 4°C, 5 min) and removal of the supernatant, the neurons were resuspended and seeded in a 24-well plate containing poly-D-lysine-coated circular coverslips at 50 μL per well. After microscopic examination of the neuronal state, 500 μL per well of neuronal culture medium was slowly placed into the plate, and the neurons were cultured in an incubator (37°C, 5% CO_2). After 1.5–2 h, the neurons were used for experiments.

Ratiometric (Ca^{2+})i measurements

The cultured DRG neurons were incubated in extracellular solution (details are provided in Supplementary Table 1) containing 1% PowerLoad concentrate (Component B of Fluo-4 Calcium Imaging Kit) and 0.1% Fluo-4 AM (Component A of Fluo-4 Calcium Imaging Kit) for 15–30 min. After removing the solution, neurons with coverslips were washed once with extracellular solution and transferred to an observation dish located on the microscope. A standard fluorescein isothiocyanate (FITC) filter was employed to detect fluorescence intensity (calcium-bound Fluo-4 dye; excitation 494 nm/emission 506 nm). Neurons were stimulated with menthol (1, 10, and 100 μM) or menthol (100 μM) with AMTB (0.1, 1, 10 μM) for 5 min. The calcium ionophore ionomycin (1 μM, Enzo Life Sciences, New York, USA) was used to ensure the viability of the cells at the end of the experiments for 1 min. F/F0 was the ratio of fluorescence intensity at each time point to the first second, and △Fmax was the maximum value of F/F0 during stimulation before the use of ionomycin. All solutions were freshly prepared. The fluorescence intensity was calculated using

ImageJ 1.53 q (NIH, USA) and analyzed using Microsoft Excel (Version 2204) and GraphPad Prism V9 (GraphPad, San Diego, CA).

Colonic crypt isolation and organoid culture

The whole colon of the C57BL/6 mice or biopsies of the normal human colon were used to isolate colonic crypts. Human colonic biopsies were collected during endoscopy from patients without colon-related diseases who consented to provide colonic biopsy specimens for research purposes. The collection was approved by the Ethics Committee of East Hospital Affiliated to Tongji University (Permit Number: 2021-017). Colonic crypts were isolated using crypt isolation buffer (10 mM EDTA; Life Technologies, Scotland, UK, in PBS) and washed twice with crypt wash buffer (0.04% BSA; Sigma-Aldrich, Steinheim, Germany) and 1% streptomycin and penicillin in Advanced DMEM/F12 (Life Technologies, Scotland, UK). The crypts were subsequently resuspended in ice-cold growth factor-reduced Matrigel (Discovery Labware, Bedford, USA) and seeded in a 24-well plate with 500 μL of organoid culture medium (IntestiCult Organoid Growth Medium (Mouse) or IntestiCult Organoid Growth Medium (Human), Cambridge, MA, USA, with 1% streptomycin and penicillin).

Substance P release from isolated mouse colon

SP release were determined by intestinal lumen dilation experiments of the mice distal colon in vitro without or with different pressures (90 mmHg and 150 mmHg) and supplemented with different stimulants (menthol 100μM or/and AMTB 10μM) to determine the effect of stimulants on SP release from distal colon under normal and nociceptive stimuli. The washed colon was wound around and fastened to acrylic rods and placed in synthetic interstitial fluid (SIF) side a thermostatic shaking bath at

37°C for 30 min for equilibration. Experiments were initiated by transferring the mounted colon into the control (SIF) tube. After 5 min the colons were moved into the second tubes (SIF or SIF with stimulants), third tubes (SIF or SIF with stimulants), forth tubes (SIF or SIF with stimulants) for 5 min subsequently. In order to promote the release of SP and mimic noxious stimuli, the colons were pressurized by 90 mmHg and 150 mmHg in the third and fourth steps, respectively. The supernatant was measured by ELISA. Acquired data were normalized to the control baseline (before stimulation), which reduced variability.

ND7/23 cells differentiation and stimulation

ND7/23 cells were purchased from Fenghui Biotechnology Co. Ltd. (CL0604, Hunan, China), and have been validated and tested for mycoplasma contamination. The Cells were cultured in Dulbecco's Modified Eagle Medium (DMEM) (Biosharp, Beijing, China) with 10% fetal bovine serum (FBS) (Thermo Fisher) and 1% penicillin/streptomycin, and were differentiated in neuronal culture medium that was also used to culture DRG. In stimulation experiments, ND7/23 cells or differentiated ND7/23 cells were stimulated with menthol 100 μM or AMTB 10 μM for 6 h in DMEM with 1% penicillin/streptomycin.

RNA isolation and Quantitative PCR

Total RNA was isolated from fresh mouse colonic tissue or DRG neurons using the FastPure Cell/Tissue Total RNA Isolation Kit V2 (Vazyme, Nanjing, China), according to the manufacturer’s instructions. cDNA was synthesized using HiScript III All-in-one RT SuperMix Perfect for qPCR (Vazyme, Nanjing, China). Quantitative PCR (qPCR) analyses were performed using a predesigned ChamQ Universal SYBR qPCR

Master Mix (Vazyme, Nanjing, China) on a LightCycler 480 System (Roche, Mannheim, Germany). To calculate the relative expression of the indicated genes, actin beta (*Actb*) was used as the reference gene, and primers were purchased from BioTNT (Shanghai, China) and are listed in Supplementary Table 2.

Immunoprecipitation

The target antibody or IgG was coupled to protein A/G agarose beads (Biolinkedin, Shanghai, China), and then specifically binds to the target protein in the cell lysate to form a complex. Shake slowly at 4°C overnight, centrifuge to collect the complex precipitate, and wash 3 times with PBS. The complex was then slowly resuspended in 2× loading buffer. Samples were boiled at 100°C for 5 min, antigens, antibodies and beads were separated, and the supernatant was used for immunoblot analysis after centrifugation.

Immunoblot

The cells were rapidly washed with PBS and lysed using RIPA Lysis Buffer (Beyotime, Shanghai, China) with 1 mM PMSF (Beyotime, Shanghai, China) and protease and phosphatase inhibitor cocktail for general use, 50X (Beyotime, Shanghai, China). The solution was collected and centrifuged at 12,000 g for 20 min. Protein concentrations were determined using bicinchoninic acid reagent (Thermo Fisher, New York, USA). The lysates were denatured, electrophoresed on a polyacrylamide gel, and transferred to a nitrocellulose membrane. After blocking with 5% skim milk for 1 h, the membranes were incubated with primary antibodies for 12 h at 4 °C, followed by secondary antibodies for 1 h. The blots were visualized using the

West Pico PLUS Chemiluminescent Substrate (Thermo Fisher, New York, USA). The antibodies used in this study are listed in Supplementary Table 3. Optical density was quantified using ImageJ 1.53q. The relative optical density was calculated as a ratio to the β-actin and based on the first day or negative control as a standard for fold change.

Immunofluorescent and apoptosis staining

Colonic organoids stimulated by SP (Merck KGaA, Darmstadt, Germany) or cells cultured on circular coverslips in 24-well plates were washed with PBS and fixed in 4% PFA for 2 h. For immunofluorescence staining, the cells were blocked using 5% donkey serum in staining solution (1% BSA and 0.5% Triton X-100 in PBS) for 1 h and incubated with primary antibody in staining solution for 12 h at 4 °C. After washing with PBS, the cells were incubated with secondary antibody in the staining solution for 1 h and washed again with PBS. Coverslips with cells were removed and fixed to the slides using DAPI Fluoromount-G Mounting Medium (Southern Biotech, Birmingham, AL). An in Situ Cell Death Detection Kit, TMR red (Roche, Mannheim, Germany), and LIVE/DEAD Cell Imaging Kit (488/570) (Thermo Fisher, New York, USA) were used for tunnel staining, according to the manufacturer's instructions. After tunnel staining, cells with coverslips were fixed using DAPI Fluoromount-G Mounting Medium. The antibodies used in this study are listed in Supplementary Table 2.

Cytokine or neuropeptide measurement

Serum from DSS colitis mice was used to measure the concentrations of TNF-α, IFN-γ, IL-23, IL-1β, IL-12, and IL-10 by enzyme-linked immunosorbent assay using commercial kits (ABclonal, Wuhan, China) (catalog numbers in Supplementary Table 4). The supernatant of DRG neurons stimulated for 6 h or synthetic interstitial fluid containing neuropeptides from intestinal lumen dilation experiments was used to measure SP concentration using enzyme-linked immunosorbent assay (commercial kits, Cayman Chemical, Ann Arbor, Michigan, USA) (catalog numbers in Supplementary Table 4). All operations were performed according to the manufacturer's instructions to ensure the accuracy of the results.

GEO data collection

The GEO dataset is a public genomics platform composed of array- and sequence-based data. Data on *TRPM8* and *TAC1* expressions in patients with IBD and normal human tissues were extracted from the GEO database (GSE38713, GSE47908, GSE13367, and GSE16879). Data on *WNT3A, FZD2, FZD3, FZD4, FZD6, FZD7, FZD9, FZD10, LRP5L, LRP5, DVL1, DVL3, GSK3B, AXIN1, AXIN2, CTNNB1, LEF1, TCF7L1,* and *TCF7* expressions in patients with UC and normal human tissues were extracted from the GEO database (GSE109142). All Log(2) expression values were analyzed using GraphPad Prism V9. Volcano plot and UMAP plot of GSE109142 were analyzed by GEO2R (https://www.ncbi.nlm.nih.gov/geo/geo2r/).

SIF buffer

SIF consisted of (in mM): 107.8 NaCl, 26.2 $NaCO_3$, 9.64 Na-gluconate, 7.6 sucrose, 5.05 glucose, 3.48 KCl, 1.67 NaH_2PO_4, 1.53 $CaCl_2$ and 0.69 $MgSO_4$, gassed with 95% O_2 and 5% CO_2 to obtain a pH of 7.4.

mRNA sequencing

Total RNA was extracted using the TRIzol reagent (Invitrogen, CA, USA) according to the manufacturer's protocol. RNA purity and quantification were evaluated using the NanoDrop 2000 spectrophotometer (Thermo Scientific, USA). RNA integrity was assessed using the Agilent 2100 Bioanalyzer (Agilent Technologies, Santa Clara, CA, USA). Then the libraries were constructed using VAHTS Universal V6 RNA-seq Library Prep Kit according to the manufacturer's instructions. The transcriptome sequencing and analysis were conducted by OE Biotech Co., Ltd. (Shanghai, China). The libraries were sequenced on an llumina Novaseq 6000 platform and 150 bp paired-end reads were generated. Raw reads for each sample were generated. Raw reads of fastq format were firstly processed using fastp and the low-quality reads were removed to obtain the clean reads. Then clean reads for each sample were retained for subsequent analyses. The clean reads were mapped to the reference genome using HISAT. FPKM of each gene was calculated and the read counts of each gene were obtained by HTSeq-count. PCA analysis were performed using R(v 3.2.0) to evaluate the biological duplication of samples.

Differential expression analysis was performed using the DESeq2. Q value < 0.05 and foldchange > 2 or foldchange < 0.5 was set as the threshold for significantly

differential expression gene (DEGs). Hierarchical cluster analysis of DEGs was performed using R (v 3.2.0) to demonstrate the expression pattern of genes in different groups and samples. The radar map of top 30 genes was drawn to show the expression of up-regulated or down-regulated DEGs using R packet grader. Based on the hypergeometric distribution, GO, KEGG pathway, Reactome and WikiPathways enrichment analysis of DEGs were performed to screen the significant enriched term using R (v 3.2.0), respectively. R (v 3.2.0) was used to draw the column diagram, the chord diagram and bubble diagram of the significant enrichment term. Gene Set Enrichment Analysis (GSEA) was performed using GSEA software. The analysis was used a predefined gene set, and the genes were ranked according to the degree of differential expression in the two types of samples. Then it is tested whether the predefined gene set was enriched at the top or bottom of the ranking list.

Supplementary Tables

Supplementary Table 1 The components of extracellular solution.

Name	Content
NaCl	145 mM
KCl	5 mM
$CaCL_2$*$2H_2O$	1.25 mM
$MgCL_2$*$6H_2O$	1 mM
Hepes	10 mM
D(+)-Glucose monohydrate	10 mM

In 1L pure water (The pH was adjusted to 7.4 by NaOH; the solution was filtered to obtain the osmolarity at 308-312 mOsm).

Supplementary Table 2 Sequences of primers for quantitative PCR.

Gene	Sequence 5′–3′			
Mouse				
Actb	F	CCTCTATGCCAACACAGT	R	AGCCACCAATCCACACAG
Tnf	F	TAGCCAGGAGGGAGAACAGA	R	CCAGTGAGTGAAAGGGACAGA
Ifng	F	TCAGGTAGTAACAGGCTGTCC	R	CATTCGGGTGTAGTCACAGTT
Il1b	F	TACATCAGCACCTCACAAGC	R	AGAAACAGTCCAGCCCATACT
Il23a	F	CAACTCTGACTGAGCCCTTAGTG	R	ATAATGGTGTCCTTGCCCTTC
Il12a	F	ACACAAGAACGAGAGTTGCCT	R	ACAGGGTCATCATCAAAGACG
Il10	F	AGTGTGTATTGAGTCTGCTGG	R	GAGAGAGGTACAAACGAGGTT
Trpm8	F	AGTGGAGTTGTTCACCGAGTGT	R	GCTTCGCAGGAGTAGACCAGTA
Tac1	F	GTTGGACTAATGGGCAAAAGA	R	GTGCGTTCAGGGGTTTATTTA
Wnt2	F	CTGTCAGCCAACACCTTAG	R	ATTCCCCTTTCAGATTCTCTC
Wnt3a	F	GAGGAATGGTCTCTCGGGAGTT	R	CATTGTTGTGACGGTTCATGG

Supplementary Table 3 Antibodies for Western blot assay and immunostaining.

Antibody	Vendor	Catalog No.
HRP anti-rabbit	Abcam	ab6721
HRP anti-β-actin	Abcam	ab49900
Alexa Fluor® 555 donkey anti-rabbit IgG (H+L)	Thermo Fisher Scientific	A31572
Alexa Fluor® 488 donkey anti-rabbit IgG (H+L)	Thermo Fisher Scientific	A32790
Anti-TRPM8	ABclonal	A12572
Anti-Substance P	ABclonal	A20772
Anti-p-p38 (Thr180/Tyr182)	Cell Signaling Technology	4511
Anti-p38	Cell Signaling Technology	9212
Anti-p-erk1/2 (Thr202/Tyr204)	Cell Signaling Technology	4370
Anti-erk1/2	Cell Signaling Technology	4695
Anti-non-p (Active) β-catenin (Ser33/37/Thr41)	Cell Signaling Technology	8814
Anti-GSK3β	ABclonal	A2081
Anti-p-GSK3β-S9	ABclonal	AP0039
Anti-PKAca	ABclonal	AP18603
Anti-p-PKAca-T197	ABclonal	AP0557

Anti-β-catenin	Cell Signaling Technology	9582
Anti-ki67	Abcam	ab16667
Rabbit Control IgG	ABclonal	AC005

Supplementary Table 4 ELISA Kit for inflammatory cytokines and neuropeptides measurement.

Name	Vendor	Catalog No.
Mouse TNF-alpha ELISA Kit	ABclonal	RK00027
Mouse IFN-gamma ELISA Kit	ABclonal	RK00019
Mouse IL-1 beta ELISA Kit	ABclonal	RK00006
Mouse IL-10 ELISA Kit	ABclonal	RK00016
Mouse IL-12 p70 ELISA Kit	ABclonal	RK00018
Mouse IL-23 ELISA Kit	ABclonal	RK00102
Substance P ELISA Kit	Cayman Chemical	583751

Original Data

Original Western Blots images 1 followed the order of appearance in the Figures.

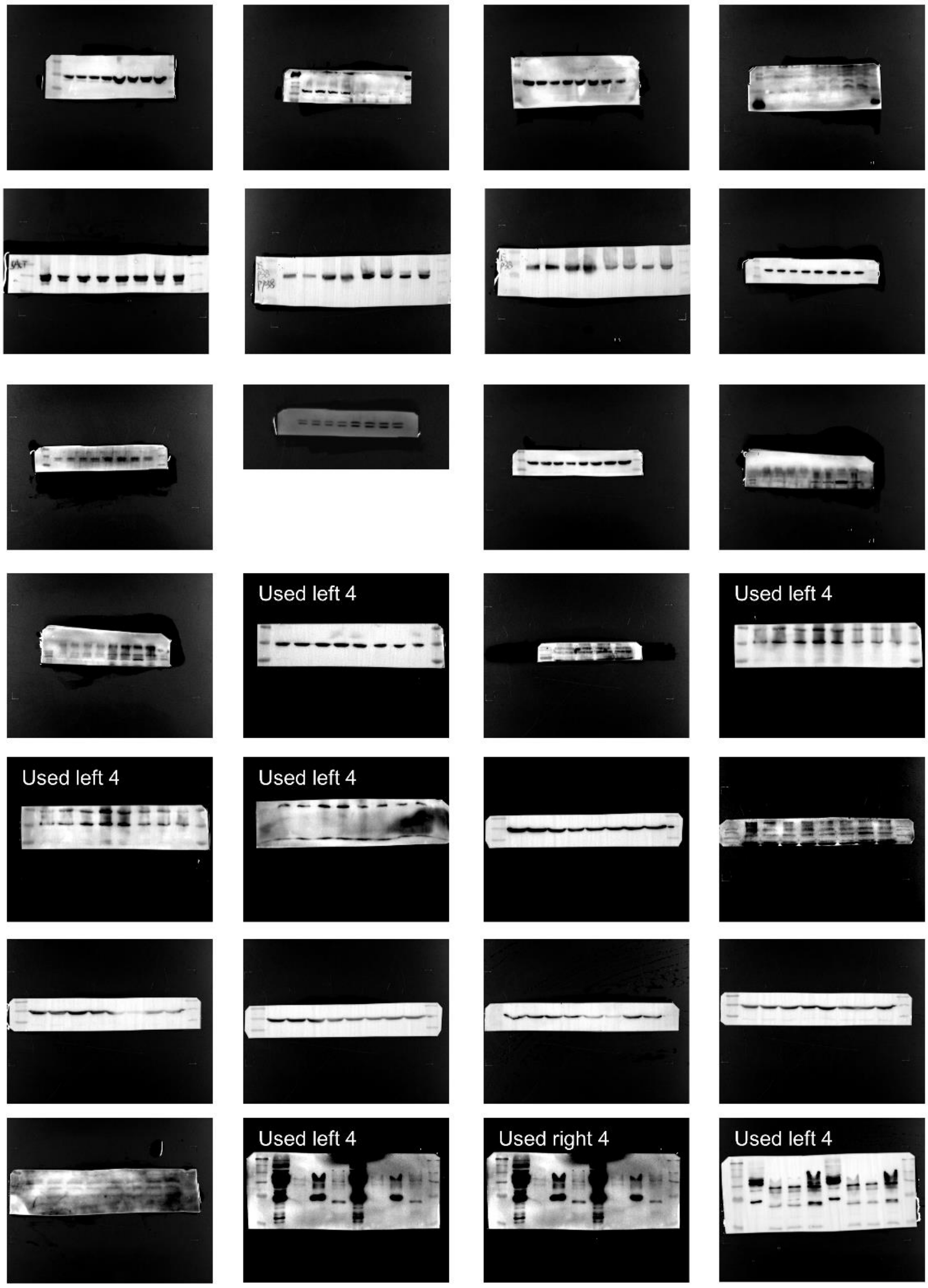

Original Western Blots images 2 followed the order of appearance in the Figures.

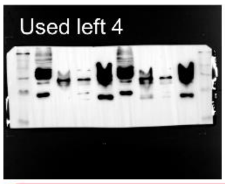

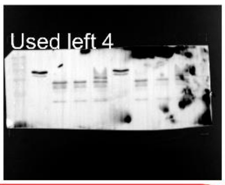

Different exposure time for the same membrane

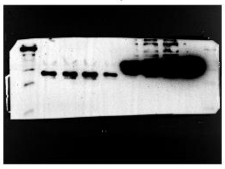

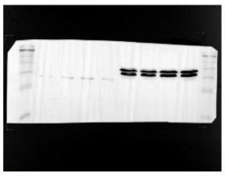

Different exposure time for the same membrane

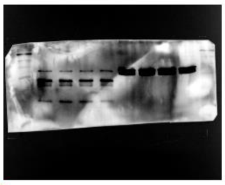

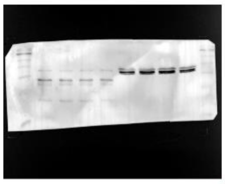

Different exposure time for the same membrane

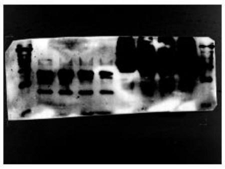

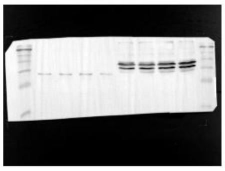

Different exposure time for the same membrane

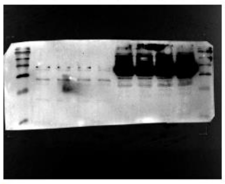

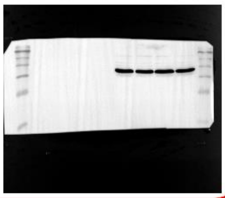

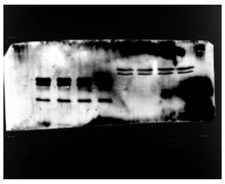

Different exposure time for the same membrane

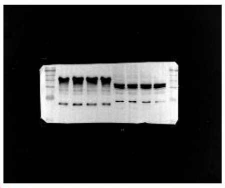

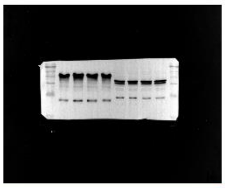

Different exposure time for the same membrane

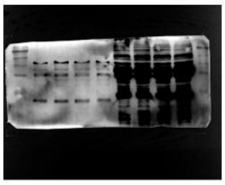

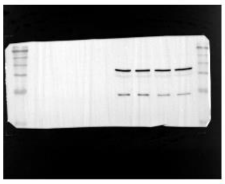

Different exposure time for the same membrane

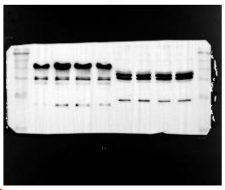

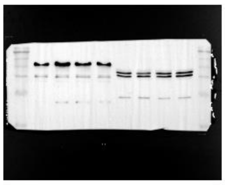

Different exposure time for the same membrane

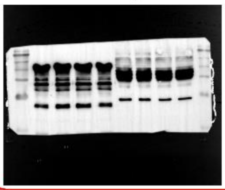

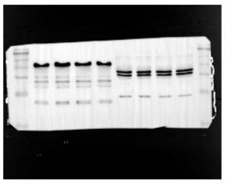

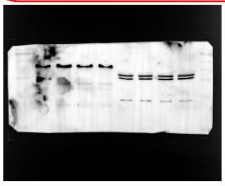

Original Western Blots images 3 followed the order of appearance in the Supplementary Figures.

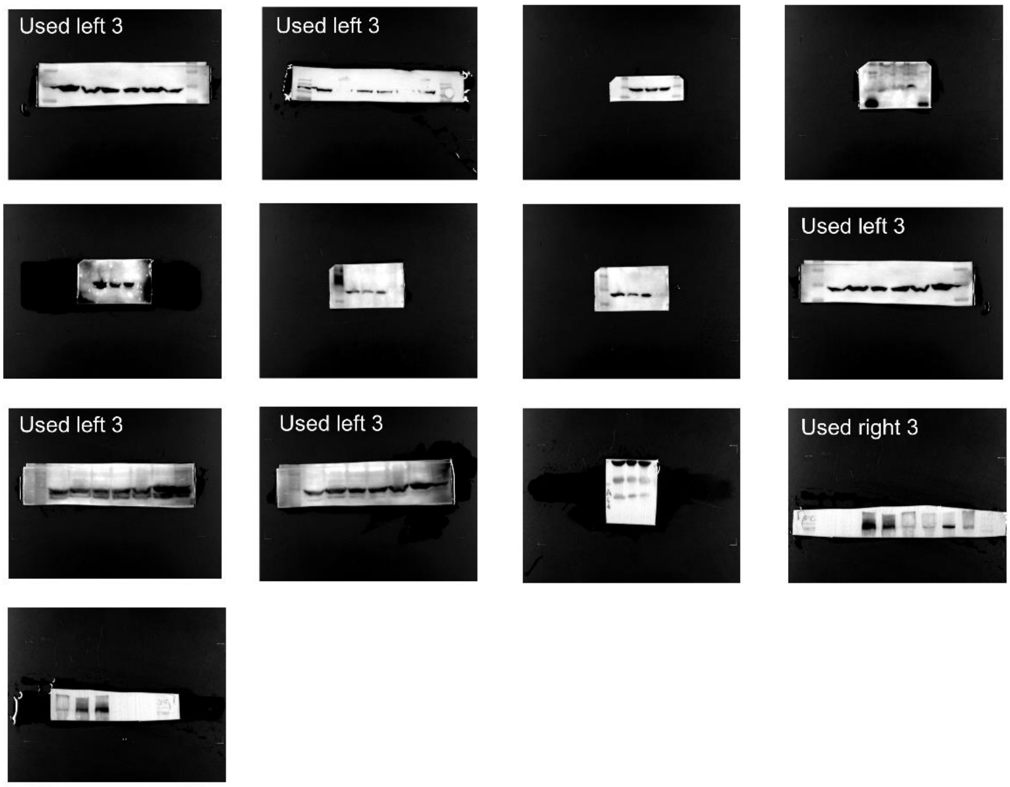

Fig.1D

DAY	NC								2.5%DSS							
**0**	100.0	100.0	100.0	100.0	100.0	100.0	100.0	100.0	100.0	100.0	100.0	100.0	100.0	100.0	100.0	100.0
**1**	98.3	101.1	101.4	102.0	101.5	107.0	102.5	103.9	102.0	100.4	101.8	103.7	102.1	102.2	104.3	101.1
**2**	99.6	99.3	101.4	101.0	102.7	106.0	103.5	101.5	105.9	102.5	99.6	101.7	102.8	100.4	101.4	98.5
**3**	101.3	100.7	102.2	101.3	103.8	107.8	105.0	101.9	96.0	99.3	101.4	100.7	100.4	98.7	97.5	93.3
**4**	103.0	102.5	101.4	103.6	102.3	103.8	104.0	101.0	89.7	89.5	98.2	95.3	96.1	94.7	96.0	91.4
**5**	102.2	102.1	101.4	102.6	102.7	101.8	106.0	102.9	86.6	83.7	92.1	88.9	87.6	87.7	87.7	86.2
**6**	103.9	104.6	104.3	104.6	105.0	102.0	107.0	103.9	81.0	79.0	87.8	86.5	81.3	83.3	81.6	82.5
**7**	103.5	107.5	104.3	105.6	106.1	102.0	104.5	101.5	77.9	74.3	82.1	84.5	73.9	75.4	75.5	79.5
**8**	105.2	106.8	104.7	104.6	107.7	106.5	107.0	103.9	72.3	68.8	78.1	80.1	70.7	69.3	72.2	74.3

Fig.1F

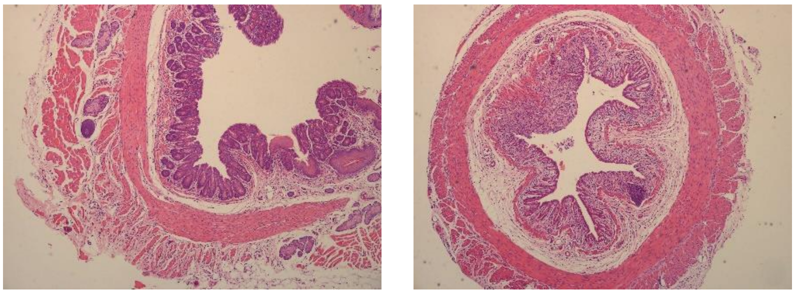

Fig.1K

DAY	Control						Menthol 100µM					AMTB 10µM					Menthol 100µM+AMTB 10µM				
**0**	100	100	100	100	100	100	100	100	100	100	100	100	100	100	100	100	100	100	100	100	100
**1**	97.3	97.3	94.9	98.1	100.0	100.4	96.5	100.7	98.9	97.8	98.1	98.7	97.3	96.4	98.5	98.1	98.5	95.1	96.9	96.5	93.3
**2**	95.0	95.0	92.2	96.6	98.2	96.1	93.7	99.3	96.4	96.3	96.2	95.7	93.3	93.2	95.2	97.8	94.8	93.6	96.5	93.7	92.3
**3**	93.9	93.9	91.0	97.4	95.2	92.1	93.7	97.4	94.3	95.2	93.9	95.3	93.3	93.2	92.2	96.2	94.4	93.6	96.1	90.6	89.2
**4**	92.7	92.7	91.0	93.2	92.3	93.4	92.1	98.9	94.6	92.3	94.3	95.3	91.4	94.0	92.2	92.5	92.5	91.0	95.7	90.2	89.9
**5**	90.0	90.0	91.0	89.1	88.2	94.3	94.1	95.9	96.8	91.5	90.8	92.7	89.4	94.8	91.1	86.8	86.6	91.0	91.3	89.0	86.5
**6**	86.2	86.2	91.0	86.4	85.6	93.4	94.9	94.5	94.6	90.4	90.1	92.3	87.8	93.6	87.4	85.6	85.8	88.0	87.0	88.6	83.5
**7**	82.8	82.8	89.8	83.8	80.4	86.9	94.9	92.6	93.2	88.9	88.9	87.6	85.5	92.0	82.9	81.8	80.6	88.0	85.0	84.3	81.5
**8**	82.0	82.0	83.9	81.9	73.8	81.2	94.9	93.4	88.9	88.9	85.5	84.1	82.4	88.8	74.3	78.1	78.0	86.9	84.3	83.1	79.1

Fig.1N

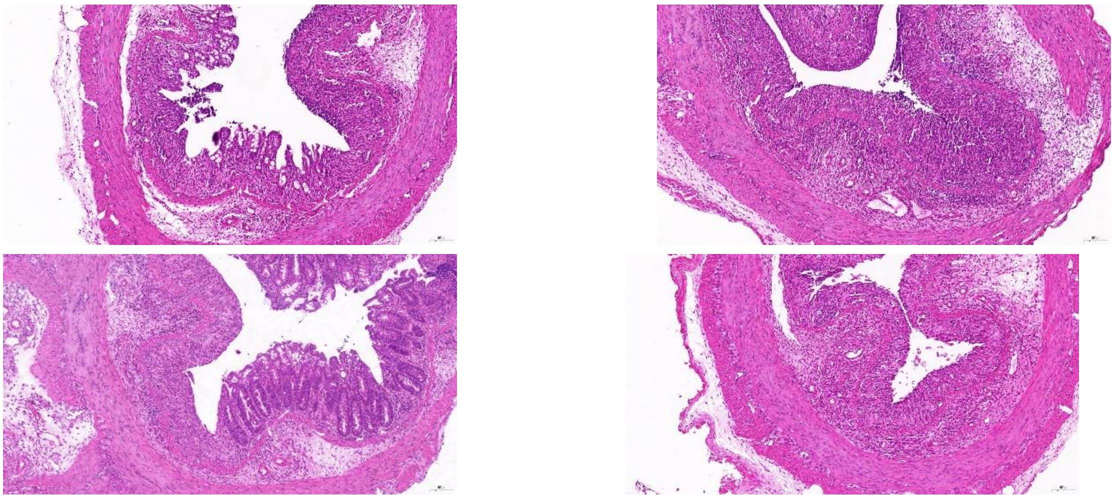

Fig.2A

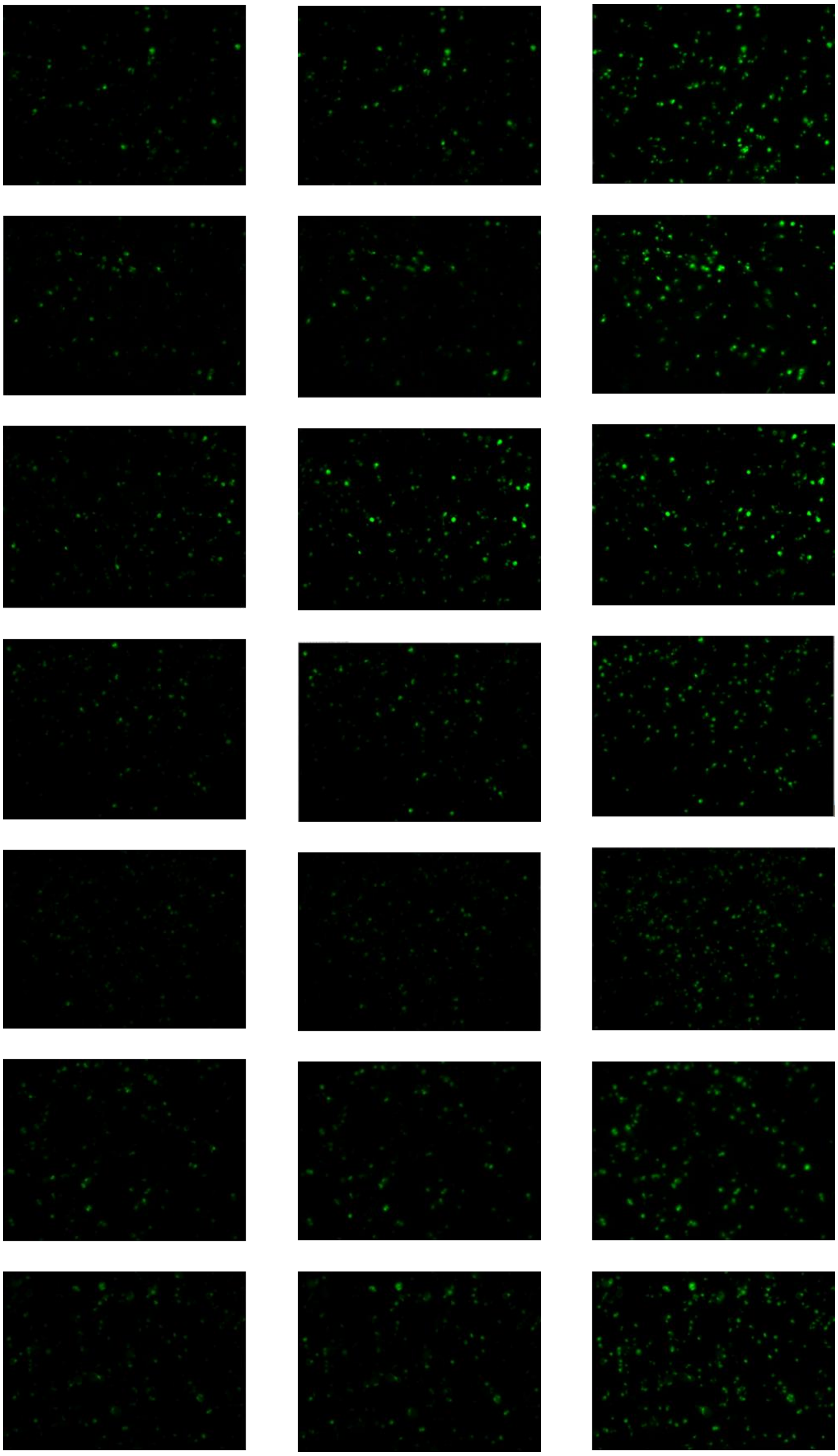

Fig.2E

Menthol 1μM	Menthol 10μM	Menthol 100μM
1.18909668	1.65332462	2.14136376
2.24451844	1.90652042	2.90922667
2.09013401	2.47707299	1.69387406
1.40618806	1.92498118	1.35443272
1.7646007	1.52887065	1.5873533
1.1494515	2.23915709	1.63462593
1.3878425	2.48017412	1.38321142
1.79538964	1.88230628	1.54747113
1.79660004	2.5114418	1.31484773
1.55170176	1.91521797	1.50545893
2.00730593	1.41894511	1.31200218
1.26076541	1.54143744	1.76171156
2.15162224	1.48419622	1.67419566
1.45962616	2.49338398	1.73262447
1.86206988	2.23994832	1.48942159
1.38628023	1.89675871	1.37370069
1.12207214	1.55635482	1.37535333
1.32228669	1.62062731	7.01221985
1.7765914	1.66937357	5.78326605
2.19180992	1.55402589	5.97286698
1.68576427	1.75261675	5.45597585
1.82121156	1.65625036	1.48904169
1.73055699	1.63157243	2.57384951
1.08019984	1.60084675	2.33690236
2.14159143	2.34570039	1.34067672
1.80687594	1.55032816	1.34545455
0.86376062	1.86341925	1.36196319
1.70403324	1.6913693	1.38461538
0.87116	3.388909	2.03703704
1.95463923	1.56194208	2.01886792
1.77229275	1.55577324	1.91428571
1.77419469	2.01216276	2.02325487
1.77272562	1.8895177	2.67646872
1.16167612	1.46849995	2.31999834
1.8711447	2.23739337	2.3799977
1.41855878	1.88770286	2.688888
2.01269047	1.815344	1.6163522
1.21455	1.88333489	1.73631715
1.048631	1.6219709	1.58257388
1.80347888	1.5759635	1.58257388
1.44733848	1.91406283	2.02173892
0.99941473	1.93096975	1.96296333
1.1769116	1.48244068	1.85714417
1.10193099	1.75104906	1.6490478
1.3489356	1.72222334	1.76
1.57092262	2.29247003	1.84090909
2.20891717	1.3928574	1.76197715
1.67070803	2.08594869	2.22179389
1.39947177	1.98014686	1.76530286

Fig.2F

Menthol 100µM	Menthol 100µM+AMTB 0.1µM	Menthol 100µM+AMTB 1µM	Menthol 100µM+AMTB 10µM
1.45996439	1.533499	1.51292576	0.94700833
1.59700116	1.43773577	1.84347004	1.17258062
1.84200707	1.31905077	1.18790453	1.26526909
1.64587732	1.37953369	1.28458935	1.16255898
1.85736231	1.38501314	1.78420546	1.01636416
1.21875018	1.24957429	1.64877007	1.21914849
2.53897039	1.24314259	1.84802937	1.42267171
1.33490566	1.46470864	1.73951297	1.53338355
1.19578328	2.36612717	1.4693726	1.56881936
1.19728161	1.44474971	1.07967038	1.31925439
1.61594256	1.36690563	1.48228426	0.95559733
1.51718389	1.31809038	1.29351243	1.23725039
1.54620982	1.50720198	1.08022621	1.29033899
1.54471692	1.37990416	1.09039503	1.09471786
1.09677589	2.25235345	1.49845823	1.22707809
1.67593911	1.41171074	1.11744521	1.08155315
1.71265696	1.8592374	1.02999651	0.93637673
1.18573789	2.01425794	1.04385835	1.26300248
1.0141154	1.21130857	1.56145741	1.19270507
1.17165796	1.62139306	1.14503869	1.22944464
1.2377074	1.83388263	1.42526938	1.10165362
18.7194482	1.49809644	1.24301136	1.26488119
5.54569881	1.82936381	1.75724761	1.35003207
10.7602526	1.33624427	1.2481322	1.2706005
20.815711	1.33912949	1.4622133	1.30469771
13.5445749	2.34941554	1.33407899	1.12625616
6.05531983	1.60356857	1.19101051	1.41256913
8.44933012	1.85915511	1.1757196	1.41030423
1.8541709	1.23131428	1.08571318	1.40285022
1.51166858	1.97585288	1.22485323	1.19195724
1.44484309	1.4444454	0.99183609	1.13880342
1.13919611	1.27711323	1.14498658	1.29431788
1.15639242	1.51351313	1.06753852	1.04360558
1.13759476	1.30787027	1.50447567	0.95505889
1.31052632	1.57312578	1.27384373	1.21372816
1.30167598	1.36433807	1.22935032	1.36247496
1.2982245	1.3212902	1.45880248	1.28553918
1.9530072	1.68484747	1.2198997	0.94082811
2.0877193	1.37674681	1.34318608	1.42540176
1.94642857	1.26037711	1.50799855	1.12422847
1.98550783	1.49999425	1.29465546	1.30895356
2.36932385	1.5081961	1.54895	1.23280496
2.45237825	3.28419422	1.42927095	1.40981594
2.23076857	1.98387667	1.50650373	1.28173502
2.65958791	1.45510311	1.80667888	1.39488584
2.44230769	1.66844347	1.78897886	1.42492845
1.69014018	2.3028503	2.0249412	1.3117792
1.78190951	1.57623235	1.84776522	1.19278185
1.95989149	1.8331431	1.84299662	1.39715439
2.10526453	1.63230314	1.64857482	1.2137954
1.96153713	1.81615492	1.13575858	1.82926577
1.93548307	1.74876708	1.48798662	1.20777298
2.25714278	2.34365357	1.11442097	1.4077181
2.09677419	2.09078625	1.79668713	1.02829058
1.71232877	1.5853392	1.32198591	1.20819753
1.70930233	1.73760679	1.74024876	1.13881933
1.72172637	1.65146897	1.78747055	1.19428148
2.23127375	2.16995778	0.92081855	0.96666772
	1.84874077	1.22346934	1.29070037
	1.58677679	1.05220575	1.24048259
	1.96856428	1.13892635	1.09058558
	1.58909426	1.08695652	0.97800357
		1.38297965	
		1.22899998	
		1.20118194	

Fig.6B

DAY	Control						SP 1μM					
**0**	1	1	1	1	1	1	1	1	1	1	1	1
**1**	1.48	1.69	1.00	1.37	2.25	1.21	1.08	1.18	1.49	1.04	1.32	1.24
**2**	1.81	2.89	1.36	1.81	2.43	1.49	1.88	2.29	2.05	1.71	2.52	1.33
**3**	2.02	2.68	1.81	2.05	3.42	1.83	1.80	2.76	2.76	2.59	1.94	2.05
**4**	2.43	3.16	2.31	2.40	4.06	2.24	2.06	3.07	2.72	2.78	2.05	2.37
**5**	3.00	3.73	3.13	2.87	4.94	2.89	1.82	2.98	2.71	3.32	2.31	1.31
**6**	3.67	4.42	3.84	2.90	5.27	3.24	1.98	3.01	2.66	2.70	2.15	1.94
**7**	5.16	4.90	4.28	2.73	6.64	3.52	1.85	3.00	2.61	3.16	2.27	2.26
**8**	5.64	5.50	4.26	3.50	6.24	4.01	2.02	2.91	2.74	2.86	2.31	2.30

Fig.6F

Ki67	Controln=91	SP 1μMn=110		TUNEL	Controln=32	SP 1μMn=24
	8	0			3	23
	5	2			3	16
	4	0			13	17
	3	2			13	15
	6	3			53	48
	3	2			2	51
	3	0			19	69
	8	2			4	0
	8	3			3	5
	14	0			3	20
	4	0			12	15
	12	1			4	26
	9	1			0	20
	7	1			2	11
	11	2			14	8
	4	0			8	9
	3	0			28	25
	12	5			5	0
	3	2			13	0
	4	0			3	13
	5	0			0	5
	4	0			2	6
	6	2			1	14
	2	1			1	18
	21	2			6	
	8	0			0	
	10	0			4	
	4	1			6	
	16	3			8	
	6	0			4	
	6	5			4	
	14	4			0	
	7	2				
	12	2				
	13	0				
	9	3				
	10	1				
	11	6				
	6	5				
	5	1				
	25	2				
	37	0				
	8	15				
	17	8				
	12	0				
	7	1				
	17	0				
	12	1				
	56	2				
	16	1				
	6	0				
	26	0				
	8	6				
	17	0				
	21	4				
	11	6				
	10	5				
	6	5				
	9	1				
	8	5				
	33	2				
	29	2				
	11	1				
	19	8				
	14	11				
	21	0				
	27	0				
	13	4				
	22	0				
	10	7				
	16	8				
	43	6				
	58	6				
	24	1				
	28	2				
	14	1				
	11	5				
	13	6				
	15	23				
	14	1				
	42	1				
	79	1				
	38	3				
	25	0				
	43	3				
	31	2				
	26	2				
	38	1				
	41	0				
	64	1				
	54	0				
		2				
		4				
		2				
		1				
		0				
		1				
		2				
		3				
		2				
		0				
		2				
		6				
		4				
		3				
		1				
		12				
		2				
		1				
		19				

Fig.7B

0h control		48h control		48h control		48h SP1uM		48h SP1uM	
序号	大小	序号	大小	序号	fold change	序号	大小	序号	fold change
Mean	68349.71	Mean	230688.00	Mean	3.38	Mean	148779.24	Mean	2.18
STABW	61777.39	STABW	210112.10	STABW	3.07	STABW	138833.90	STABW	2.03
n	270.00	n	416.00	n	416.00	n	453.00	n	453.00
SEM	3759.65	SEM	10301.59	SEM	0.15	SEM	6522.99	SEM	0.10
1	115644	1	195740	1	2.86	1	114152	1	1.67
2	362253	2	472240	2	6.91	2	104550	2	1.53
3	48364	3	81952	3	1.20	3	171736	3	2.51
4	160140	4	107364	4	1.57	4	273258	4	4.00
5	38988	5	38200	5	0.56	5	100898	5	1.48
6	71559	6	216562	6	3.17	6	153350	6	2.24
7	84618	7	293958	7	4.30	7	148676	7	2.18
8	26202	8	30820	8	0.45	8	60932	8	0.89
9	40152	9	19072	9	0.28	9	445056	9	6.51
10	33054	10	127818	10	1.87	10	129300	10	1.89
11	26790	11	41894	11	0.61	11	147318	11	2.16
12	177600	12	20718	12	0.30	12	152896	12	2.24
13	56979	13	19042	13	0.28	13	374122	13	5.47
14	59502	14	332990	14	4.87	14	72614	14	1.06
15	32826	15	79724	15	1.17	15	197018	15	2.88
16	100521	16	34882	16	0.51	16	361280	16	5.29
17	125568	17	50232	17	0.73	17	108116	17	1.58
18	80049	18	207866	18	3.04	18	76608	18	1.12
19	79380	19	57304	19	0.84	19	45536	19	0.67
20	49725	20	81238	20	1.19	20	85218	20	1.25
21	41349	21	88414	21	1.29	21	64968	21	0.95
22	30159	22	32096	22	0.47	22	107902	22	1.58
23	194781	23	202910	23	2.97	23	64148	23	0.94
24	32562	24	274558	24	4.02	24	129182	24	1.89
25	41007	25	52554	25	0.77	25	290494	25	4.25
26	27651	26	167130	26	2.45	26	22312	26	0.33
27	53562	27	216358	27	3.17	27	101486	27	1.48
28	69435	28	118344	28	1.73	28	109404	28	1.60
29	29520	29	202536	29	2.96	29	145256	29	2.13
30	21483	30	73612	30	1.08	30	81464	30	1.19
31	18654	31	454080	31	6.64	31	137532	31	2.01
32	53175	32	125410	32	1.83	32	524948	32	7.68
33	130792	33	337530	33	4.94	33	58238	33	0.85
34	28716	34	74844	34	1.10	34	86184	34	1.26
35	16413	35	50292	35	0.74	35	131430	35	1.92
36	66135	36	63700	36	0.93	36	50314	36	0.74
37	9072	37	64894	37	0.95	37	135766	37	1.99
38	24420	38	45090	38	0.66	38	67176	38	0.98

39	97167	39	95570	39	1.40	39	1022366	39	14.96
40	23247	40	33680	40	0.49	40	112034	40	1.64
41	44808	41	348004	41	5.09	41	391460	41	5.73
42	20241	42	248972	42	3.64	42	44558	42	0.65
43	93699	43	89278	43	1.31	43	107990	43	1.58
44	186288	44	433106	44	6.34	44	166564	44	2.44
45	29104	45	1031024	45	15.08	45	192520	45	2.82
46	75831	46	161402	46	2.36	46	354934	46	5.19
47	67251	47	136860	47	2.00	47	189912	47	2.78
48	65328	48	550372	48	8.05	48	176896	48	2.59
49	113980	49	226662	49	3.32	49	341382	49	4.99
50	84121	50	518492	50	7.59	50	58848	50	0.86
51	404019	51	76220	51	1.12	51	94046	51	1.38
52	128040	52	124990	52	1.83	52	275292	52	4.03
53	66954	53	301708	53	4.41	53	625564	53	9.15
55	28206	54	38504	54	0.56	54	180798	54	2.65
56	44272	55	23332	55	0.34	55	95844	55	1.40
57	380530	56	24528	56	0.36	56	420582	56	6.15
58	91720	57	412398	57	6.03	57	450488	57	6.59
59	95456	58	224268	58	3.28	58	644894	58	9.44
60	279598	59	125434	59	1.84	59	52412	59	0.77
61	32488	60	73896	60	1.08	60	114592	60	1.68
62	150640	61	143426	61	2.10	61	102610	61	1.50
63	30942	62	107948	62	1.58	62	128064	62	1.87
64	96264	63	33200	63	0.49	63	64328	63	0.94
65	178014	64	77932	64	1.14	64	63038	64	0.92
66	52220	65	20372	65	0.30	65	112590	65	1.65
67	100166	66	47738	66	0.70	66	285534	66	4.18
68	44878	67	79624	67	1.16	67	162464	67	2.38
69	175420	68	132376	68	1.94	68	274216	68	4.01
70	27104	69	589962	69	8.63	69	53476	69	0.78
71	42152	70	165980	70	2.43	70	173924	70	2.54
72	59234	71	680538	71	9.96	71	74390	71	1.09
73	26114	72	184552	72	2.70	72	69352	72	1.01
74	49952	73	51978	73	0.76	73	49244	73	0.72
75	23650	74	118124	74	1.73	74	86906	74	1.27
76	45736	75	504028	75	7.37	75	77318	75	1.13
77	173606	76	94768	76	1.39	76	227510	76	3.33
79	48124	77	248094	77	3.63	77	42976	77	0.63
80	113128	78	270800	78	3.96	78	298614	78	4.37
81	42694	79	52542	79	0.77	79	61332	79	0.90
83	220510	80	146208	80	2.14	80	62428	80	0.91
84	64948	81	65948	81	0.96	81	159128	81	2.33
85	113298	82	140098	82	2.05	82	34582	82	0.51
86	154622	83	45796	83	0.67	83	96596	83	1.41
87	60418	84	50196	84	0.73	84	304814	84	4.46
88	30432	85	135450	85	1.98	85	280292	85	4.10
89	50420	86	83046	86	1.22	86	138224	86	2.02

90	85634	87	72004	87	1.05	87	86470	87	1.27
91	106362	88	52822	88	0.77	88	58946	88	0.86
92	64886	89	30392	89	0.44	89	184066	89	2.69
93	12108	90	107956	90	1.58	90	123764	90	1.81
94	21556	91	61716	91	0.90	91	102788	91	1.50
95	16968	92	124466	92	1.82	92	63924	92	0.94
96	32644	93	51654	93	0.76	93	156416	93	2.29
97	37544	94	193508	94	2.83	94	223656	94	3.27
98	35176	95	447976	95	6.55	95	28630	95	0.42
99	37048	96	194630	96	2.85	96	47838	96	0.70
100	43298	97	206066	97	3.01	97	41066	97	0.60
101	53664	98	593080	98	8.68	98	363078	98	5.31
102	27904	99	1036404	99	15.16	99	73738	99	1.08
103	29388	100	214690	100	3.14	100	370596	100	5.42
104	94634	101	116142	101	1.70	101	504230	101	7.38
105	9996	102	58858	102	0.86	102	90752	102	1.33
106	30072	103	149760	103	2.19	103	73606	103	1.08
107	26452	104	78456	104	1.15	104	397868	104	5.82
108	30790	105	227156	105	3.32	105	86422	105	1.26
109	57874	106	489768	106	7.17	106	56210	106	0.82
110	30604	107	174940	107	2.56	107	147902	107	2.16
111	60788	108	89738	108	1.31	108	247312	108	3.62
112	102550	109	159422	109	2.33	109	238524	109	3.49
113	13488	110	106504	110	1.56	110	90944	110	1.33
114	31582	111	124056	111	1.82	111	160334	111	2.35
115	41458	112	79846	112	1.17	112	70196	112	1.03
116	44272	113	182566	113	2.67	113	218636	113	3.20
117	380530	114	164766	114	2.41	114	73858	114	1.08
118	91720	115	126388	115	1.85	115	50148	115	0.73
119	95456	116	198692	116	2.91	116	84782	116	1.24
120	279598	117	413650	117	6.05	117	43874	117	0.64
121	32488	118	86976	118	1.27	118	105432	118	1.54
122	150640	119	136556	119	2.00	119	119620	119	1.75
123	30942	120	74352	120	1.09	120	126712	120	1.85
124	96264	121	176872	121	2.59	121	238812	121	3.49
125	178014	122	101360	122	1.48	122	374376	122	5.48
126	52220	123	135074	123	1.98	123	237336	123	3.47
127	100166	124	406862	124	5.95	124	63714	124	0.93
128	44878	125	159570	125	2.33	125	311866	125	4.56
129	175420	126	97574	126	1.43	126	89124	126	1.30
130	27104	127	149328	127	2.18	127	147686	127	2.16
131	42152	128	181026	128	2.65	128	22096	128	0.32
132	59234	129	66094	129	0.97	129	80576	129	1.18
133	26114	130	140694	130	2.06	130	302184	130	4.42
134	49952	131	171364	131	2.51	131	60906	131	0.89
135	23650	132	244824	132	3.58	132	54506	132	0.80
136	45736	133	164528	133	2.41	133	67616	133	0.99
137	173606	134	159648	134	2.34	134	111780	134	1.64

138	48124	135	259130	135	3.79	135	51462	135	0.75
139	220510	136	261272	136	3.82	136	207834	136	3.04
140	64948	137	233754	137	3.42	137	115224	137	1.69
141	113298	138	100246	138	1.47	138	29204	138	0.43
142	154622	139	85280	139	1.25	139	165786	139	2.43
143	60418	140	688030	140	10.07	140	44036	140	0.64
144	30432	141	424894	141	6.22	141	129284	141	1.89
145	50420	142	127456	142	1.86	142	44640	142	0.65
146	85634	143	161552	143	2.36	143	216496	143	3.17
147	106362	144	240154	144	3.51	144	650160	144	9.51
148	64886	145	236808	145	3.46	145	150874	145	2.21
149	12108	146	116256	146	1.70	146	48998	146	0.72
150	21556	147	138250	147	2.02	147	75480	147	1.10
151	16968	148	150968	148	2.21	148	249842	148	3.66
152	32644	149	177014	149	2.59	149	128464	149	1.88
153	37544	150	113656	150	1.66	150	157012	150	2.30
154	35176	151	396514	151	5.80	151	55660	151	0.81
155	37048	152	42132	152	0.62	152	52324	152	0.77
156	43298	153	263634	153	3.86	153	173142	153	2.53
157	53664	154	102682	154	1.50	154	58422	154	0.85
158	27904	155	121442	155	1.78	155	38810	155	0.57
159	29388	156	141916	156	2.08	156	79812	156	1.17
160	94634	157	527422	157	7.72	157	50584	157	0.74
161	9996	158	115258	158	1.69	158	137888	158	2.02
162	113128	159	251138	159	3.67	159	91532	159	1.34
163	42694	160	254368	160	3.72	160	40818	160	0.60
164	30072	161	414248	161	6.06	161	250492	161	3.66
165	26452	162	447166	162	6.54	162	67800	162	0.99
166	30790	163	346940	163	5.08	163	21166	163	0.31
167	57874	164	436234	164	6.38	164	127692	164	1.87
168	30604	165	18818	165	0.28	165	65004	165	0.95
169	60788	166	21304	166	0.31	166	53250	166	0.78
170	102550	167	35244	167	0.52	167	115616	167	1.69
171	13488	168	17854	168	0.26	168	144716	168	2.12
172	31582	169	643624	169	9.42	169	61148	169	0.89
173	41458	170	118686	170	1.74	170	24004	170	0.35
174	110520	171	133676	171	1.96	171	186284	171	2.73
175	28516	172	193784	172	2.84	172	232108	172	3.40
176	21784	173	65586	173	0.96	173	44730	173	0.65
177	87546	174	121958	174	1.78	174	199052	174	2.91
178	16600	175	1077794	175	15.77	175	89888	175	1.32
179	53606	176	537988	176	7.87	176	82076	176	1.20
180	47302	177	52786	177	0.77	177	323206	177	4.73
181	88896	178	79568	178	1.16	178	70724	178	1.03
182	153644	179	77984	179	1.14	179	20854	179	0.31
183	87824	180	45364	180	0.66	180	67324	180	0.98
184	197186	181	67560	181	0.99	181	94146	181	1.38
185	67294	182	18890	182	0.28	182	413222	182	6.05

186	120518	183	162276	183	2.37	183	90550	183	1.32
187	12098	184	537302	184	7.86	184	55322	184	0.81
188	10966	185	142328	185	2.08	185	68978	185	1.01
189	169596	186	190822	186	2.79	186	141888	186	2.08
190	28000	187	237560	187	3.48	187	464188	187	6.79
191	37156	188	37818	188	0.55	188	257728	188	3.77
192	21936	189	324912	189	4.75	189	52780	189	0.77
193	33276	190	147180	190	2.15	190	43988	190	0.64
194	22786	191	133696	191	1.96	191	50130	191	0.73
195	43428	192	353496	192	5.17	192	75210	192	1.10
196	56436	193	254674	193	3.73	193	147356	193	2.16
197	57528	194	437076	194	6.39	194	40968	194	0.60
198	51192	195	246036	195	3.60	195	49066	195	0.72
199	15756	196	670566	196	9.81	196	143462	196	2.10
200	93262	197	109888	197	1.61	197	26906	197	0.39
201	113016	198	90734	198	1.33	198	606216	198	8.87
202	69130	199	165562	199	2.42	199	202130	199	2.96
203	29296	200	113102	200	1.65	200	129820	200	1.90
204	95050	201	112340	201	1.64	201	73718	201	1.08
205	12626	202	61284	202	0.90	202	143872	202	2.10
206	57500	203	30566	203	0.45	203	111110	203	1.63
207	38256	204	35050	204	0.51	204	187150	204	2.74
208	30162	205	181738	205	2.66	205	146192	205	2.14
209	9460	206	204544	206	2.99	206	58814	206	0.86
210	87754	207	690140	207	10.10	207	203590	207	2.98
211	46246	208	251428	208	3.68	208	33544	208	0.49
212	39270	209	62934	209	0.92	209	74090	209	1.08
213	34662	210	187022	210	2.74	210	95178	210	1.39
214	35110	211	76910	211	1.13	211	40982	211	0.60
215	71950	212	82514	212	1.21	212	190962	212	2.79
216	86108	213	154334	213	2.26	213	69322	213	1.01
217	69306	214	488070	214	7.14	214	292682	214	4.28
218	104158	215	470510	215	6.88	215	139388	215	2.04
219	39606	216	189296	216	2.77	216	37692	216	0.55
220	73070	217	105528	217	1.54	217	30156	217	0.44
221	144818	218	256222	218	3.75	218	151388	218	2.21
222	90594	219	64332	219	0.94	219	582986	219	8.53
223	17514	220	186170	220	2.72	220	860244	220	12.59
224	73360	221	92486	221	1.35	221	352380	221	5.16
225	35896	222	172552	222	2.52	222	49526	222	0.72
226	25306	223	249490	223	3.65	223	81850	223	1.20
227	73694	224	740922	224	10.84	224	133974	224	1.96
228	65096	225	235828	225	3.45	225	301412	225	4.41
229	18916	226	202420	226	2.96	226	194342	226	2.84
230	10182	227	529772	227	7.75	227	274230	227	4.01
231	56420	228	614654	228	8.99	228	43976	228	0.64
232	104288	229	1175004	229	17.19	229	102014	229	1.49
233	27044	230	542210	230	7.93	230	147538	230	2.16

234	109880	231	101582	231	1.49	231	239142	231	3.50
235	22844	232	495020	232	7.24	232	299352	232	4.38
236	33662	233	924324	233	13.52	233	35920	233	0.53
237	42774	234	192400	234	2.81	234	106318	234	1.56
238	83316	235	424840	235	6.22	235	179068	235	2.62
239	50322	236	534748	236	7.82	236	254616	236	3.73
240	64526	237	296428	237	4.34	237	33338	237	0.49
241	16306	238	92930	238	1.36	238	279870	238	4.09
242	41860	239	782848	239	11.45	239	138252	239	2.02
243	141896	240	121724	240	1.78	240	214864	240	3.14
244	37852	241	170262	241	2.49	241	66380	241	0.97
245	84072	242	125510	242	1.84	242	70074	242	1.03
246	20140	243	328218	243	4.80	243	48412	243	0.71
247	29234	244	727406	244	10.64	244	345864	244	5.06
248	32122	245	98272	245	1.44	245	220464	245	3.23
249	29526	246	304138	246	4.45	246	41132	246	0.60
250	29884	247	211016	247	3.09	247	26018	247	0.38
251	33622	248	76606	248	1.12	248	68594	248	1.00
252	12000	249	227122	249	3.32	249	152418	249	2.23
253	54024	250	209566	250	3.07	250	205182	250	3.00
254	58336	251	252368	251	3.69	251	260648	251	3.81
255	107404	252	399232	252	5.84	252	583278	252	8.53
256	16996	253	113740	253	1.66	253	42524	253	0.62
257	11464	254	474020	254	6.94	254	196554	254	2.88
258	59938	255	89886	255	1.32	255	274404	255	4.01
259	39296	256	174874	256	2.56	256	215368	256	3.15
260	6040	257	131472	257	1.92	257	472690	257	6.92
261	140484	258	120144	258	1.76	258	392246	258	5.74
262	86658	259	56070	259	0.82	259	73462	259	1.07
263	40390	260	37852	260	0.55	260	61118	260	0.89
264	56018	261	157780	261	2.31	261	48736	261	0.71
265	86796	262	324932	262	4.75	262	49970	262	0.73
266	64588	263	470024	263	6.88	263	112798	263	1.65
267	9210	264	143192	264	2.09	264	22436	264	0.33
268	41428	265	447416	265	6.55	265	524380	265	7.67
269	194870	266	137866	266	2.02	266	97288	266	1.42
270	95560	267	495336	267	7.25	267	73450	267	1.07
271	54230	268	49318	268	0.72	268	39056	268	0.57
272	27756	269	81006	269	1.19	269	130498	269	1.91
273	14062	270	201774	270	2.95	270	32792	270	0.48
		271	503328	271	7.36	271	92922	271	1.36
		272	103748	272	1.52	272	51704	272	0.76
		273	325450	273	4.76	273	522210	273	7.64
		274	295942	274	4.33	274	48906	274	0.72
		275	146864	275	2.15	275	237308	275	3.47
		276	50728	276	0.74	276	274962	276	4.02
		277	160812	277	2.35	277	42418	277	0.62
		278	270882	278	3.96	278	429818	278	6.29

279	776282	279	11.36	279	38552	279	0.56
280	357092	280	5.22	280	138996	280	2.03
281	245436	281	3.59	281	66388	281	0.97
282	366680	282	5.36	282	75090	282	1.10
283	38082	283	0.56	283	102052	283	1.49
284	616634	284	9.02	284	86308	284	1.26
285	654276	285	9.57	285	154184	285	2.26
286	213632	286	3.13	286	27834	286	0.41
287	309908	287	4.53	287	85714	287	1.25
288	784762	288	11.48	288	85742	288	1.25
289	303876	289	4.45	289	389446	289	5.70
290	122882	290	1.80	290	73692	290	1.08
291	85142	291	1.25	291	108390	291	1.59
292	143510	292	2.10	292	80004	292	1.17
293	166580	293	2.44	293	75894	293	1.11
294	233578	294	3.42	294	510796	294	7.47
295	121800	295	1.78	295	92304	295	1.35
296	267256	296	3.91	296	140260	296	2.05
297	72982	297	1.07	297	92958	297	1.36
298	109886	298	1.61	298	70304	298	1.03
299	107948	299	1.58	299	104526	299	1.53
300	81716	300	1.20	300	58808	300	0.86
301	207320	301	3.03	301	155012	301	2.27
302	461144	302	6.75	302	34964	302	0.51
303	133518	303	1.95	303	52536	303	0.77
304	353758	304	5.18	304	94174	304	1.38
305	484348	305	7.09	305	176870	305	2.59
306	77702	306	1.14	306	32152	306	0.47
307	153966	307	2.25	307	620438	307	9.08
308	607728	308	8.89	308	190806	308	2.79
309	195762	309	2.86	309	40414	309	0.59
310	605530	310	8.86	310	58424	310	0.85
311	155334	311	2.27	311	165592	311	2.42
312	297724	312	4.36	312	76396	312	1.12
313	104116	313	1.52	313	45448	313	0.66
314	330310	314	4.83	314	141294	314	2.07
315	165614	315	2.42	315	56658	315	0.83
316	105418	316	1.54	316	48916	316	0.72
317	69608	317	1.02	317	273000	317	3.99
318	1079758	318	15.80	318	224640	318	3.29
319	118776	319	1.74	319	57926	319	0.85
320	129046	320	1.89	320	92450	320	1.35
321	222668	321	3.26	321	148870	321	2.18
322	203924	322	2.98	322	110094	322	1.61
323	633006	323	9.26	323	81872	323	1.20
324	122820	324	1.80	324	299476	324	4.38
325	692580	325	10.13	325	157092	325	2.30
326	186100	326	2.72	326	478150	326	7.00

327	476372	327	6.97	327	109694	327	1.60
328	68352	328	1.00	328	234756	328	3.43
329	81856	329	1.20	329	47100	329	0.69
330	172104	330	2.52	330	73974	330	1.08
331	284504	331	4.16	331	64814	331	0.95
332	251924	332	3.69	332	237096	332	3.47
333	35998	333	0.53	333	48202	333	0.71
334	46052	334	0.67	334	44780	334	0.66
335	354126	335	5.18	335	113050	335	1.65
336	777204	336	11.37	336	96272	336	1.41
337	111468	337	1.63	337	27644	337	0.40
338	173164	338	2.53	338	298820	338	4.37
339	105178	339	1.54	339	54638	339	0.80
340	260214	340	3.81	340	150666	340	2.20
341	59472	341	0.87	341	94782	341	1.39
342	154960	342	2.27	342	90258	342	1.32
343	134228	343	1.96	343	84090	343	1.23
344	34218	344	0.50	344	138904	344	2.03
345	153322	345	2.24	345	206846	345	3.03
346	128428	346	1.88	346	181274	346	2.65
347	493756	347	7.22	347	65482	347	0.96
348	157224	348	2.30	348	107376	348	1.57
349	355500	349	5.20	349	85028	349	1.24
350	402616	350	5.89	350	462692	350	6.77
351	357054	351	5.22	351	68372	351	1.00
352	211702	352	3.10	352	26904	352	0.39
353	249300	353	3.65	353	85410	353	1.25
354	226254	354	3.31	354	151108	354	2.21
355	175240	355	2.56	355	37894	355	0.55
356	30398	356	0.44	356	144610	356	2.12
357	246834	357	3.61	357	89630	357	1.31
358	351876	358	5.15	358	56628	358	0.83
359	622180	359	9.10	359	57800	359	0.85
360	176198	360	2.58	360	21672	360	0.32
361	83234	361	1.22	361	185072	361	2.71
362	434490	362	6.36	362	246424	362	3.61
363	694614	363	10.16	363	281600	363	4.12
364	177748	364	2.60	364	113686	364	1.66
365	365616	365	5.35	365	92846	365	1.36
366	180906	366	2.65	366	81546	366	1.19
367	140572	367	2.06	367	77116	367	1.13
368	351844	368	5.15	368	425428	368	6.22
369	823808	369	12.05	369	319142	369	4.67
370	417808	370	6.11	370	122124	370	1.79
371	189430	371	2.77	371	34896	371	0.51
372	119142	372	1.74	372	46738	372	0.68
373	214140	373	3.13	373	197664	373	2.89
374	58146	374	0.85	374	97916	374	1.43

375	48300	375	0.71	375	92806	375	1.36
376	243572	376	3.56	376	117736	376	1.72
377	79276	377	1.16	377	149908	377	2.19
378	70200	378	1.03	378	574786	378	8.41
379	51106	379	0.75	379	67736	379	0.99
380	199476	380	2.92	380	489710	380	7.16
381	310134	381	4.54	381	25400	381	0.37
382	420358	382	6.15	382	52140	382	0.76
383	96742	383	1.42	383	247704	383	3.62
384	1045298	384	15.29	384	379840	384	5.56
385	79752	385	1.17	385	187114	385	2.74
386	34006	386	0.50	386	850192	386	12.44
387	371242	387	5.43	387	75834	387	1.11
388	438726	388	6.42	388	69128	388	1.01
389	129336	389	1.89	389	104128	389	1.52
390	36888	390	0.54	390	103512	390	1.51
391	571374	391	8.36	391	31126	391	0.46
392	132220	392	1.93	392	253140	392	3.70
393	197628	393	2.89	393	176688	393	2.59
394	176576	394	2.58	394	75686	394	1.11
395	291598	395	4.27	395	237042	395	3.47
396	144216	396	2.11	396	282676	396	4.14
397	44384	397	0.65	397	55024	397	0.81
398	789486	398	11.55	398	105996	398	1.55
399	501050	399	7.33	399	46384	399	0.68
400	1146596	400	16.78	400	31096	400	0.45
401	69500	401	1.02	401	187304	401	2.74
402	88496	402	1.29	402	42532	402	0.62
403	186706	403	2.73	403	324364	403	4.75
404	96034	404	1.41	404	44692	404	0.65
405	209238	405	3.06	405	43936	405	0.64
406	60494	406	0.89	406	71952	406	1.05
407	85112	407	1.25	407	61808	407	0.90
408	255824	408	3.74	408	149510	408	2.19
409	65420	409	0.96	409	233218	409	3.41
410	65528	410	0.96	410	185942	410	2.72
411	33306	411	0.49	411	103302	411	1.51
412	42294	412	0.62	412	173952	412	2.55
413	58444	413	0.86	413	355534	413	5.20
414	189346	414	2.77	414	67596	414	0.99
415	309086	415	4.52	415	35356	415	0.52
416	382788	416	5.60	416	163982	416	2.40
				417	112694	417	1.65
				418	61802	418	0.90
				419	65270	419	0.95
				420	58364	420	0.85
				421	68468	421	1.00
				422	71928	422	1.05

423	39956	423	0.58
424	166808	424	2.44
425	25230	425	0.37
426	42176	426	0.62
427	23948	427	0.35
428	66616	428	0.97
429	51898	429	0.76
430	63902	430	0.93
431	18346	431	0.27
432	49412	432	0.72
433	31800	433	0.47
434	38172	434	0.56
435	23880	435	0.35
436	50790	436	0.74
437	59724	437	0.87
438	75294	438	1.10
439	82690	439	1.21
440	57348	440	0.84
441	22650	441	0.33
442	33090	442	0.48
443	53592	443	0.78
444	243346	444	3.56
445	96184	445	1.41
446	100610	446	1.47
447	367512	447	5.38
448	91416	448	1.34
449	81750	449	1.20
450	38838	450	0.57
451	75498	451	1.10
452	206684	452	3.02
453	117246	453	1.72

Fig.7D

MITTELWERT	49.9338843	MITTELWERT	20.47692308
STABW	45.12403933	STABW	22.09570985
n	121	n	130
SEM	4.102185394	SEM	1.937921951
control. 48h		SP 1μM. 48h	
NO.		NO.	
1	111	1	11
2	28	2	4
3	24	3	6
4	35	4	11
5	126	5	4
6	14	6	23
7	100	7	14
8	67	8	90
9	110	9	10
10	13	10	38
11	138	11	28
12	72	12	64
13	63	13	2
14	41	14	18
15	44	15	30
16	34	16	14
17	78	17	19
18	89	18	55
19	15	19	6
20	29	20	22
21	20	21	27
22	7	22	24
23	32	23	17
24	41	24	14
25	23	25	18
26	81	26	15
27	46	27	62
28	57	28	10
29	56	29	0
30	41	30	7
31	39	31	41
32	57		24
33	4		8
34	20		4
35	110		39
36	44		12
37	17		8
38	66		8
39	167		20

40	32	31
41	29	54
42	20	125
43	12	18
44	47	8
45	8	10
46	146	1
47	44	78
48	7	8
49	19	69
50	10	29
51	18	30
52	137	16
53	4	3
54	121	25
55	186	11
56	85	3
57	35	25
58	42	61
59	41	3
60	7	12
61	98	23
62	21	21
63	101	22
64	42	1
65	37	22
66	173	36
67	100	18
68	28	10
69	121	13
70	53	19
71	9	6
72	36	1
73	8	62
74	90	17
75	22	2
76	148	9
77	4	5
78	24	18
79	8	11
80	55	2
81	130	1
82	62	1
83	51	1
84	7	33
85	22	18
86	105	7
87	22	31

88	1	7
89	43	40
90	7	13
91	4	7
92	8	4
93	91	6
94	9	16
	54	22
	17	23
	90	3
	110	13
	70	1
	19	4
	2	87
	8	0
	16	5
	4	45
	6	105
	4	50
	4	34
	27	11
	62	9
	75	20
	27	5
	9	3
	97	2
	74	31
	31	2
	12	5
	9	0
	30	6
	30	1
	10	14
	196	10
		43
		18
		4
		7
		51
		32
		13
		19
		14

Fig.7F

MITTELWERT	4.546341463	MITTELWERT	11.42931937
STABW	7.371384044	STABW	13.33156046
n	205	n	191
SEM	0.514839794	SEM	0.96463786
control. 48h		SP 1μM. 48h	
NO.		NO.	
1	18	1	3
2	4	2	3
3	7	3	5
4	30	4	4
5	81	5	1
6	3	6	3
7	3	7	9
8	8	8	6
9	19	9	15
10	11	10	3
11	20	11	3
12	3	12	1
13	4	13	12
14	5	14	10
15	2	15	2
16	5	16	9
17	8	17	21
18	4	18	1
19	9	19	5
20	2	20	1
21	2	21	13
22	1	22	11
23	0	23	10
24	1	24	0
25	3	25	15
26	2	26	8
27	5	27	6
28	11	28	3
29	9	29	3
30	2	30	9
31	0	31	8
32	0		13
33	9		9
34	8		9
35	5		6
36	0		8
37	14		9
38	0		26
39	1		3

40	0	11
41	3	4
42	4	23
43	0	4
44	4	5
45	5	8
46	0	6
47	1	2
48	3	4
49	0	14
50	0	16
51	11	12
52	5	23
53	0	3
54	3	84
55	0	4
56	1	2
57	2	1
58	5	3
59	11	5
60	6	3
61	0	6
62	15	22
63	8	41
64	17	14
65	2	0
66	15	4
67	4	9
68	2	19
69	0	13
70	1	12
71	2	13
72	5	1
73	11	1
74	16	46
75	0	7
76	0	11
77	7	9
78	6	42
79	1	0
80	1	0
81	0	0
82	1	8
83	0	5
84	3	6
85	0	21
86	0	33
87	13	45

88	0	6
89	1	5
90	8	2
91	2	43
92	5	4
93	6	3
94	7	2
95	12	8
96	7	10
97	6	49
98	0	31
99	8	0
100	9	0
101	0	0
102	0	0
103	1	1
104	4	0
105	5	22
106	0	5
107	3	3
108	4	9
109	2	3
110	5	8
111	5	2
112	0	12
113	8	6
114	11	7
115	3	3
116	3	15
117	13	7
118	0	21
119	1	10
120	0	14
121	0	16
122	0	8
123	0	7
124	8	24
125	0	4
126	0	9
127	3	26
128	1	19
129	3	9
130	0	12
131	2	18
132	1	32
133	12	25
134	5	21
135	3	14

136	0	19
137	0	8
138	11	7
139	0	16
140	0	14
141	0	1
142	1	27
143	0	3
144	0	98
145	0	25
146	1	10
147	3	14
148	0	9
149	0	3
150	4	48
151	11	7
152	0	26
153	3	10
154	4	9
155	4	4
156	6	28
157	0	16
158	8	3
159	0	10
160	0	5
161	0	7
162	1	16
163	2	34
164	12	4
165	1	15
166	1	19
167	6	31
168	3	2
169	0	5
170	2	6
171	11	7
172	0	3
173	0	1
174	0	0
175	1	4
176	3	41
177	6	0
178	0	5
179	0	10
180	4	0
181	2	1
182	1	2
183	5	4

184	11	21
185	2	12
186	0	1
187	4	8
188	1	14
189	7	18
190	7	6
191	1	0
192	6	
193	0	
194	0	
195	3	
196	12	
197	0	
198	2	
199	0	
200	7	
201	21	
202	6	
203	2	
204	10	
205	23	

Fig.8B

	Control				
	体重（%）				
Day	1	2	3	4	5
0	100	100	100	100	100
1	98.09886	97.66537	97.0696	104.878	103.4483
2	100.7605	101.9455	98.5348	102.8455	103.8793
3	101.9011	100.7782	99.6337	104.4715	103.0172
4	103.0418	102.3346	98.1685	106.0976	106.4655
5	101.1407	103.1128	98.9011	106.9106	104.7414
6	102.6616	100	100.7326	104.4715	106.8966
7	103.8023	100.7782	97.4359	107.7236	108.1897
	103.4221	101.9455	101.4652	106.9106	106.0345

	Menthol 100μM				
	体重（%）				
Day	1	2	3	4	5
0	100	100	100	100	100
1	97.2549	101.2605	102.6667	99.27798	97.59036
2	99.21569	102.9412	101.3333	104.6931	99.19679
3	100.7843	103.7815	103.5556	101.8051	101.2048
4	98.43137	105.8824	104.4444	102.8881	99.59839
5	100.3922	105.8824	105.3333	101.444	102.4096
6	102.3529	105.042	104.8889	104.3321	101.6064
7	103.1373	103.7815	107.1111	105.0542	103.2129
	103.9216	105.4622	106.2222	106.4982	104.4177

	Aprepitant 3mg/kg				
	体重（%）				
Day	1	2	3	4	5
0	100	100	100	100	100
1	99.32203	101.6598	102.0325	98.4375	101.1765
2	100.678	100.8299	102.439	102.3438	102.7451
3	101.6949	102.9046	100.813	103.5156	103.5294
4	102.3729	103.7344	103.6585	100.7813	104.3137
5	101.3559	104.9793	103.252	103.9063	102.7451
6	103.0508	102.0747	105.2846	105.4688	105.8824
7	100.678	104.5643	105.6911	105.4688	106.6667
	100	104.5643	106.0976	107.8125	105.8824

	Menthol 100μM+Aprepitant 3mg/kg				
	体重（%）				
Day	1	2	3	4	5
0	100	100	100	100	100
1	100.3984	101.2821	99.61977	102.9167	98.34711

2	100.3984	102.9915	101.9011	102.0833	99.58678
3	101.1952	101.2821	101.1407	104.5833	98.76033
4	102.3904	102.5641	102.6616	102.9167	101.2397
5	104.7809	104.2735	101.9011	105.8333	99.17355
6	105.1793	105.5556	104.5627	108.75	98.34711
7	102.3904	106.8376	104.1825	107.9167	99.17355
	105.9761	107.265	103.4221	108.75	100.4132

DSS

体重（%）

Day	1	2	3	4	5
0	100	100	100	100	100
1	100	101.8182	98.83268	99.30314	101.8868
2	97.83394	102.9091	103.1128	100.3484	102.6415
3	98.55596	103.2727	102.3346	100.3484	101.8868
4	97.83394	97.09091	98.83268	97.56098	100
5	93.14079	90.90909	91.43969	93.37979	92.07547
6	90.61372	86.54545	87.93774	90.94077	87.54717
7	84.83755	79.63636	83.26848	90.94077	78.86792
	74.00722	72	77.43191	87.45645	70.9434

DSS+Menthol

100μM

体重（%）

Day	1	2	3	4	5
0	100	100	100	100	100
1	100.738	102.8674	100.3953	101.3793	102.381
2	101.107	102.8674	101.1858	100	100.7937
3	100.738	105.3763	101.9763	100.6897	101.5873
4	101.107	101.0753	98.81423	100	99.20635
5	103.321	99.28315	90.51383	99.65517	97.22222
6	102.214	98.56631	83.79447	99.65517	96.03175
7	98.89299	91.75627	78.65613	100.6897	92.06349
	95.20295	87.81362	67.98419	101.7241	80.95238

DSS+Aprepitant 3mg/kg

体重（%）

Day	1	2	3	4	5
0	100	100	100	100	100
1	100.6667	101.2048	101.9531	98.14815	101.1765
2	103.3333	102.4096	102.3438	102.7778	102.7451
3	102	105.2209	102.7344	104.1667	103.5294
4	99.66667	103.6145	99.60938	100.9259	100.3922
5	93.66667	100.4016	95.3125	100	98.82353
6	89.33333	96.38554	93.35938	97.22222	98.03922
7	87	95.18072	89.84375	97.22222	94.90196

	80.66667	97.18876	82.42188	100	82.35294

DSS+Menthol 100μM+Aprepitant 3mg/kg
体重（%）

Day	1	2	3	4	5
0	100	100	100	100	100
1	100.3831	101.0949	99.18033	98.92857	103.4483
2	100.3831	98.90511	101.6393	98.21429	104.3103
3	101.1494	101.0949	100.8197	100.3571	103.0172
4	98.46743	98.54015	97.54098	98.92857	105.6034
5	95.01916	96.35036	97.54098	97.85714	106.8966
6	93.48659	95.62044	96.31148	96.78571	105.1724
7	90.8046	93.06569	91.80328	93.21429	104.7414
	86.59004	95.25547	90.98361	93.21429	102.1552

Fig.8F

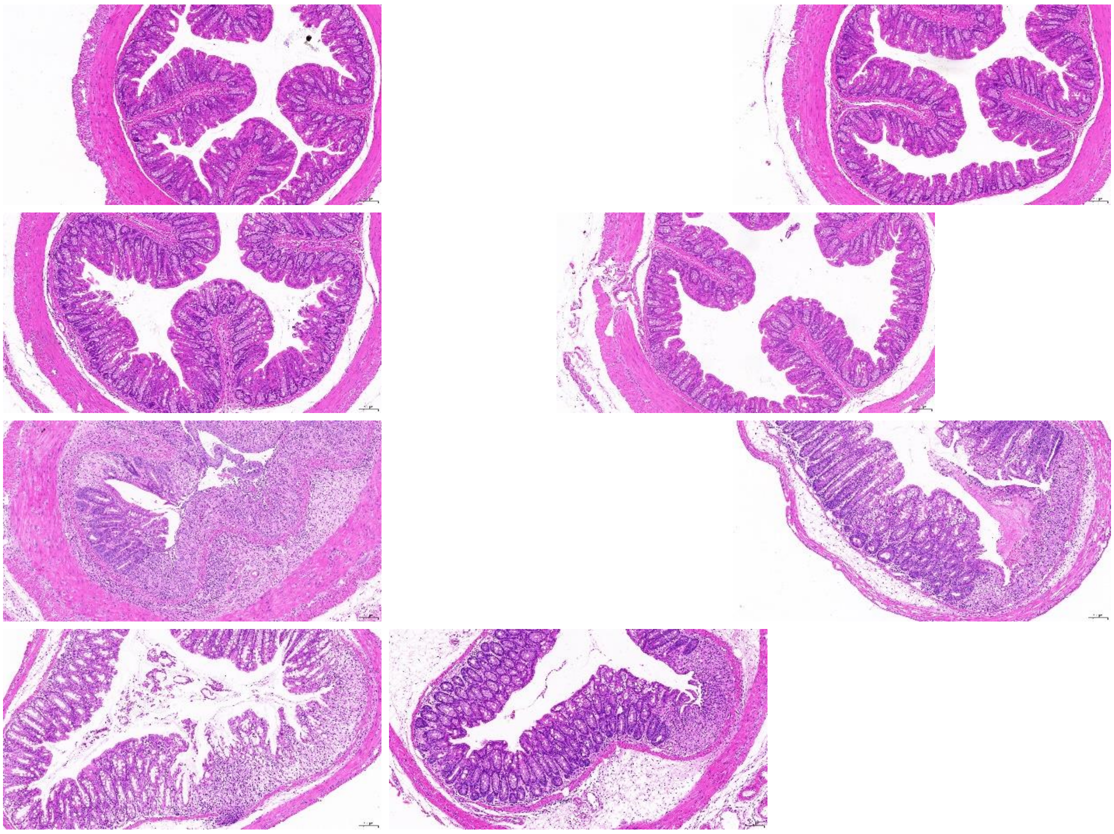

Some scatter plots can directly represent the original data values and will not be mentioned here.
